# Large-scale fabrication of highly ordered sub-20 nm noble metal nanoparticles on silica substrates without metallic adhesion layers

**DOI:** 10.1038/s41378-017-0001-2

**Published:** 2018-04-23

**Authors:** Hai Le-The, Erwin Berenschot, Roald M. Tiggelaar, Niels R. Tas, Albert van den Berg, Jan C. T. Eijkel

**Affiliations:** 1 BIOS Lab-on-a-Chip Group, MESA+ Institute for Nanotechnology, MIRA Institute for Biomedical Technology and Technical Medicine, Max Planck Center for Complex Fluid Dynamics, University of Twente, Enschede, 7522 NB The Netherlands; 2 Mesoscale Chemical Systems Group, MESA+ Institute for Nanotechnology, University of Twente, Enschede, 7522 NB The Netherlands; 3 NanoLab Cleanroom, MESA+ Institute for Nanotechnology, University of Twente, Enschede, 7522 NB The Netherlands

## Abstract

Periodic noble metal nanoparticles offer a wide spectrum of applications including chemical and biological sensors, optical devices, and model catalysts due to their extraordinary properties. For sensing purposes and catalytic studies, substrates made of glass or fused-silica are normally required as supports, without the use of metallic adhesion layers. However, precise patterning of such uniform arrays of silica-supported noble metal nanoparticles, especially at sub-100 nm in diameter, is challenging without adhesion layers. In this paper, we report a robust method to large-scale fabricate highly ordered sub-20 nm noble metal nanoparticles, i.e., gold and platinum, supported on silica substrates without adhesion layers, combining displacement Talbot lithography (DTL) with dry-etching techniques. Periodic photoresist nanocolumns at diameters of ~110 nm are patterned on metal-coated oxidized silicon wafers using DTL, and subsequently transferred at a 1:1 ratio into anti-reflection layer coating (BARC) nanocolumns with the formation of nano-sharp tips, using nitrogen plasma etching. These BARC nanocolumns are then used as a mask for etching the deposited metal layer using inclined argon ion-beam etching. We find that increasing the etching time results in cone-shaped silica features with metal nanoparticles on the tips at diameters ranging from 100 nm to sub-30 nm, over large areas of 3×3 cm^2^. Moreover, subsequent annealing these sub-30 nm metal nanoparticle arrays at high-temperature results in sub-20 nm metal nanoparticle arrays with ~10^10^ uniform particles.

## Nanotechnology: Fabrication method for large-scale arrays of noble metal nanoparticles

A versatile nanofabrication method has been developed for patterning large-scale arrays of noble metal nanoparticles supported on oxidized silicon substrates without the need for metallic adhesion layers. Thanks to their remarkable properties and diverse applications, nanoparticles made from noble metals have attracted enormous interest. For many purposes, the nanoparticles require glass or fused silica substrates as support without the use of metallic adhesion layers. However, precise patterning of such nanoparticles is difficult without adhesion layers—especially with nanoparticles under 100 nm in diameter. A team headed by Hai Le-The at the University of Twente, Netherlands succeeded in developing a robust method for large-scale fabrication of highly ordered noble metal nanoparticles under 20-nm diameter supported on silica substrates without adhesion layers. The authors believe their method has wide potential in biological and chemical applications.

## Introduction

Noble metal nanoparticles (NPs) have been studied intensively due to their widespread applications in both academic research and industry. The reduction in size of noble metal particles, for example gold (Au) and platinum (Pt) particles, to the nanoscale results in extraordinary properties, especially optical and catalytic properties^1,2^. The most compelling optical property of metallic NPs is their localized surface plasmon resonance (LSPR)^3^. When excited by incident light, the conduction electrons in metallic NPs are stimulated to collectively oscillate at a resonant frequency, thus absorbing the incident light at a specific wavelength. By manipulating this LSPR property of metallic NPs, especially in an ordered arrangement, various applications have been demonstrated including optical devices^4^, chemical and biological sensors^5,6^, fuel and solar cells^7,8^, and surface-enhanced Raman spectroscopy (SERS)^9^, showing the great benefits of using noble metal-NP arrays. Besides having excellent optical properties for sensing purposes, noble metallic NPs at diameters of a few or tens of nanometers have also shown enormously high catalytic activity. Haruta et al. first observed that supported Au nanoparticles at the diameter of ~5 nm possess extremely high catalytic activity for the oxidation of carbon monoxide even at temperatures far below 0 °C^10^. The particle size and shape, composition, oxidation state, and the interaction of the particle with its support are attributed to strongly affect the catalytic activity simultaneously^11^. However, under chemical reactions, metallic NPs have found to be unstable due to the sintering of particles which results in blocking of active sites and deactivation^12^. Therefore, for catalysis studies, highly ordered and uniform metallic NPs on support substrates are highly demanded as better control in the uniformity and distribution of the metallic NPs leads to better controlled properties. Such arrays of supported metallic NPs can serve as model systems to investigate their thermal stability and catalytic properties^13^. Therefore, the aim of this study is to fabricate arrays of supported noble metal nanoparticles with diameters of several tens of nanometers that can be used for gas-phase catalysis studies.

Although having a large number of applications as outlined above, large-scale uniform arrays of noble metal NPs with tunable sizes down to 20 nm and supported on ceramic substrates such as glass, fused-silica or oxidized-silicon, are difficult to fabricate, especially without the use of metallic adhesion layers. For top–down fabrication of Au and Pt nanostructures, a metallic adhesion layer, i.e., titanium (Ti) or chromium (Cr), is normally required to improve their adhesion to the substrates, preventing the removal of the structures^14^. However, such adhesion layers could result in undesired reactions with Au and Pt, i.e., interdiffusion and alloying, and thus affect their structural and optical properties^15,16^. Many techniques that have been utilized to pattern such arrays can be classified into two main categories that are direct patterning techniques and template-assisted techniques. Conventional direct patterning techniques such as electron beam lithography (EBL)^17^, ion-beam lithography (IBL)^18^, and dip pen lithography^19,20^ provide opportunities to precisely control the particle size and shape, and their interspacing. However, these techniques require dedicated systems which are costly expensive, and thus not widely accessible. Moreover, their use of serial patterning limits their throughput at a relatively low yield, making them not suitable for mass production of large-area arrays. Another direct patterning technique, laser interference lithography (LIL), allows high-yield patterning of large footprint arrays^21^. However, this technique requires a high stability of the operation system in order to obtain reproducible fabrication. Template-assisted techniques are based on the idea of using templates such as block copolymers^22,23^, shadow masks^24^, nanoimprint molds^25^, and monolayer of polystyrene spheres^26^ for patterning metal nanoparticle arrays. Using such templates, arrays of metallic NPs can be fabricated through self-assembly^27^, or by metal deposition methods such as evaporation, sputtering, and electrochemical deposition^28^. These techniques, however, come with their own drawbacks. Self-assembled block copolymers are normally difficult to precisely control at the nanoscale over large areas. The use of shadow masks requires additional steps for making the masks, which are time-consuming. Moreover, significant efforts are needed in order to pattern metal nanoparticle arrays over centimeter-scale areas because of the fragility of the mask. The use of nanoimprint mold suffers from its contact with the substrate, which can lead to the significant degradation of the mold over time. Self-assembly of polystyrene spheres, on the other hand, allows very little control over particle orientation and geometric pattern variation.

Recently, an alternative top–down nanopatterning technique has been reported by Solak et al., termed displacement Talbot lithography (DTL). It allows for rapid fabrication of highly ordered nanostructures at the wafer scale, i.e., periodic photoresist patterns of lines, holes or dots, with a high yield^29^. However, when DTL is used with a monochromatic UV beam (365 nm wavelength), only photoresist nanostructures with feature sizes of a few hundreds of nanometers can be fabricated, which can be utilized as a mask for fabricating metal nanoparticle arrays. In fact, patterning arrays of metal nanoparticles with diameters below 50 nm is still challenging.

In this paper, we report and demonstrate a robust fabrication method that allows rapid patterning of highly ordered noble metal (Au and Pt) nanoparticles supported on oxidized silicon substrates, without the need of metallic adhesion layers. Our fabrication method combines UV-based DTL with subsequent plasma and ion-beam etching techniques, enabling us to fabricate 3×3 cm^2^ arrays of Au and Pt nanoparticles with diameters in the range from sub-20 nm to 100 nm, supported on cone-shaped silica features.

## Materials and methods

### Wet thermal oxidation of silicon wafers as support substrates

For all fabrication processes, silicon wafers with a thick thermal oxide layer of ~1.1 μm were used as support substrates for the metal nanoparticles. They were prepared by the wet thermal oxidation of conventional (100) 4-inch silicon (Si) wafers (525 μm thick, Okmetic, Finland). All the Si-wafers were completely cleaned before running the wet thermal oxidation process to prevent cross-contamination. In detail, Si-wafers were immersed in a 99% nitric acid (HNO_3_) solution for 10 min, and in a 69% HNO_3_ solution at 95 °C for 10 min. These Si-wafers were then rinsed with deionized (DI) water using a quick dump rinser. Subsequently, Si-wafers were immersed in a 1% hydrofluoric acid (HF) solution to remove the native oxide, rinsed in DI water again, and spin-dried. The Si-wafers were then loaded into a high-temperature tube furnace (Model 287, TEMPRESS), using a quartz carrier to implement the wet oxidation at 1150 °C for 2 h and 40 min. During the oxidation process, the flow rate of a mixture of water vapor and nitrogen gas was fixed at 2 l min^-1^. The ramping and cooling rates were set at 10 °C min^-1^ and 7 °C min^-1^, respectively.

### Patterning periodic BARC nanocolumns with nano-sharp tips

**Fig. 1:**
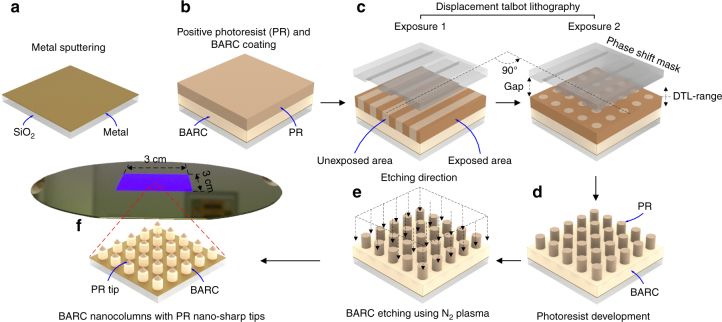
Fabrication process of periodic BARC nanocolumns with nano-sharp tips, supported on a metal-coated oxidized Si-wafer. **a** A metal layer was sputtered on an oxidized Si-wafer. **b–d** Periodic photoresist nanocolumns were patterned by DTL, and **e** subsequently etched in N_2_ plasma to directly transfer to **f** BARC nanocolumns with PR nano-sharp tips

Figure 1 shows the fabrication process of periodic BARC nanocolumns on a metal-coated oxidized Si-wafer. Briefly, a thin metal layer made of gold or platinum was deposited on the oxidized Si-wafer using an ion-beam sputtering system (home-built T’COathy system, MESA+, NanoLab). The sputtering processes were performed at 200 W, and a pressure of 6.6 × 10^−3^ mbar, which was adjusted using an argon (Ar) flow. Periodic nanocolumns at a diameter of ~110 nm were patterned by using UV-based DTL (PhableR 100C, EULITHA) in a photoresist (PR) layer of 200 ± 1.5 nm (PFI88 photoresist diluted 1:1 with propylene glycol methyl ether acetate (PGMEA), Sumitomo Chemical Co., Ltd.), and subsequently transferred at a 1:1 ratio into a bottom anti-reflection layer coating (BARC) layer of 187 ± 2 nm (AZ BARLi II 200) by using nitrogen (N_2_) plasma etching^30^. The plasma etching of BARC was conducted using a reactive ion etch (RIE) system (home-built TEtske system, MESA+, NanoLab) at wafer-level, 13 mTorr, and 25 W for 8 min.

### Argon ion-beam etching for patterning metal nanoparticle arrays

To further process the deposited metal layer, argon ion-beam etching (IBE) was performed in an etching system (IBE, Oxford i300) at 5 s.c.c.m. Ar, 300 eV, and 50–55 mA. We first investigated the etching rate of individual layers of different materials, i.e., SiO_2_, PR, BARC, Au, and Pt, in this IBE system at different beam incident angles. Thicker layers of PR and BARC were used for this investigation of the etching rate. PR layers (299.3 ± 1.8 nm) and BARC layers (245.8 ± 1.5 nm) were spin-coated on oxidized Si-wafers at 2000 r.p.m. for 45 s, followed by baking at 90 °C and 185 °C, respectively. Au and Pt layers were also deposited on oxidized Si-wafers, using the T’COathy system at 6.6 × 10^−3^ mbar, and 200 W for 2 min. The etching rate at each etching angle was determined by comparing the thickness of the initial layer with that of the layer etched for 3 min. The thicknesses of the PR and BARC layers were determined from the images taken using a high-resolution scanning electron microscope (HR-SEM, FEI Sirion microscope) at a 5 kV acceleration voltage and a spot size of 3. The thicknesses of the metal layers and the thermal oxide layers were measured using an ellipsometer system (M-2000UI, J.A. Woollam Co.) at an angle of 75°. The surface roughness was determined from atomic-force microscopy (AFM) images (scan field: 500 × 500 nm^2^), recorded in contact modes using an AFM system (Dimension Icon, Bruker Corp.) in air.

**Fig. 2:**
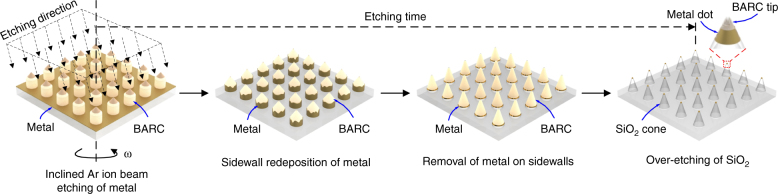
Patterning an array of metal nanoparticles supported on cone-shaped silica features. A metal-coated oxidized Si-wafer with patterned BARC nanocolumns was inclined-etched by ion-beam etching. Increasing the etching time resulted in a silica-supported metal nanoparticle array

For fabricating the metal nanoparticle arrays, the metal-coated wafers with the patterned BARC nanocolumns were subsequently inclined etched in the IBE system (Oxford i300) at 5 s.c.c.m. Ar, 300 eV, and 50–55 mA (Fig. 2). The etching time was increased with an etching time-step of 3 min, and the structure was regularly checked using the HR-SEM after every etching time-step. Overetching the SiO_2_ substrate resulted in metal nanoparticles supported on cone-shaped silica features.

## Results and discussion

### Periodic BARC nanocolumns with nano-sharp tips

**Fig. 3:**
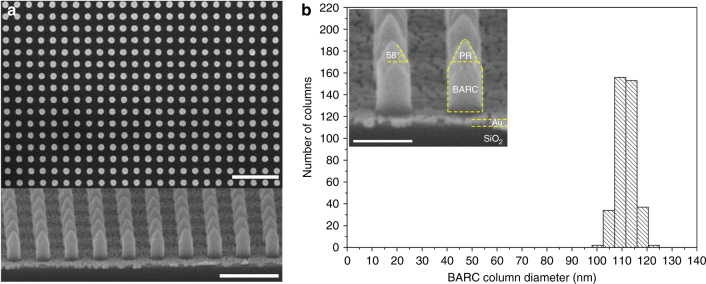
**a** Top-view (top, scale bar: 1 μm) and cross-sectional (bottom, scale bar: 500 nm) HR-SEM images of periodic BARC nanocolumns fabricated on a gold coated oxidized Si-wafer, and **b** the corresponding column diameter distribution with a close-up image inserted (scale bar: 200 nm)

Figure 3a shows the HR-SEM images of periodic BARC nanocolumns fabricated on a gold coated oxidized Si-wafer. The cross-sectional HR-SEM image shows successful fabrication with highly vertical BARC sidewalls as well as formation of PR nano-sharp tips. We attribute the tip formation to the physical bombardment of high energy particles during N_2_ plasma etching. For resist material, a dependence of the etching rate on the angle of ion incidence was reported, in which the highest etching rate occurred at an angle around 50°–60°^31^. Therefore, etching the PR nanocolumns at normal ion incidence causes facet formation at an angle directly corresponding to the angle of the maximum etching rate, resulting in the formation of PR nano-sharp tips. The close-up image clearly shows the BARC nanocolumns with the PR nano-sharp tips at an angle of 58 ± 2° (Fig. 3b). It is highly remarkable that the BARC nanocolumns with the PR nano-sharp tips—called BARC nanocolumns—were found to have an extremely high uniformity in the column height over the entire area of 3×3 cm^2^, namely 251.4 ± 1.5 nm. The top-view HR-SEM image shows a lower, but still high uniformity in the diameter (110 ± 3 nm) and periodicity of 250 nm (Fig. 3a). The narrow distribution in the diameter indicates a highly controllable fabrication process (Fig. 3b). The uniformity of the BARC nanocolumns over the entire area of 3 × 3 cm^2^ was also investigated by repeating the measurement at five selected areas within the array. Well-defined and highly ordered BARC nanocolumns with a high uniformity in the height and diameter were obtained over the large area of 3×3 cm^2^ (Supplementary Figures S1–S3 and Table S1).

### Sputtering and optimization of Au and Pt thin films

In the close-up image (Fig. 3b), a discontinuous Au layer was observed. We attribute this to the insufficient thickness of ~8.9 nm of the deposited Au layer in the sputtering system at 6.6 × 10^−3^ mbar, and 200 W for 10 s. Although a longer sputtering time results in a continuous Au layer, a thicker deposited Au layer leads to an increase in size and volume of the fabricated Au nanoparticles. Moreover, an increase in metal layer thickness could enlarge the surface roughness (Ra), caused by the increase in the Au crystallographic grain size^32^. This is also the case for sputtered Pt^32^. A large surface roughness of the deposited metal layers could lead to non-uniform arrays of metal nanoparticles. Therefore, Au and Pt layers with optimized thicknesses are needed to achieve uniform metal nanoparticle arrays. We thus investigated the thickness and surface roughness of metal layers deposited at various sputtering times in the T’COathy system at 6.6 × 10^−3^ mbar, and 200 W.

**Fig. 4:**
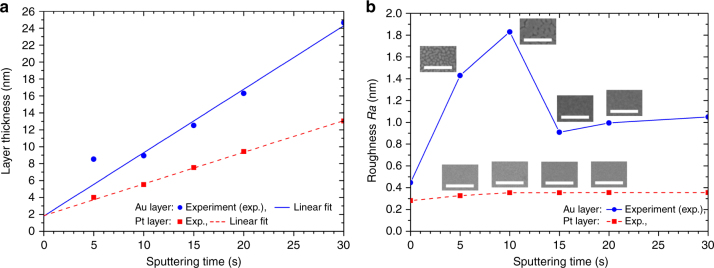
**a** Thickness of deposited metal layers and **b** their corresponding surface roughness versus sputtering time. Inserted top-view HR-SEM images in **b** (scale bar: 200 nm) show the morphological appearance of the deposited metal layers at various sputtering times

Figure 4a shows the measured thickness of the deposited metal layers as function of the sputtering time. From the linear fit curves, the sputtering rates of Au and Pt could be determined as 45.0 nm min^−1^ and 22.5 nm min^−1^, respectively. At a 5 s sputtering time, a large variation in the layer thickness between the measured value and the linear fit curve was observed. We attribute this difference to the inaccuracy of the ellipsometer system at this Au layer thickness. At this sputtering time Au nanoclusters formed on the substrates, and such discontinuous layer (Fig. 4b) can lead to an inaccurate thickness measurement. It is furthermore clearly observed in Fig. 4a that there was an offset thickness in our sputtering system, i.e. for sputtering time of 0 s the linear fit does not pass the origin. This could result from the metal deposition during the pre-sputtering process for cleaning the target, or an unexpectedly high sputtering rate when opening the shutter between the target and the substrate.

Figure 4b shows the average surface roughness (Ra) of the deposited metal layers measured over an area of 500 × 500 nm^2^ (Supplementary Figure S4–S5). Up to 10 s sputtering time, the surface roughness of the Au layers was high, which we explain by the formation of Au nanoclusters, growing over time. A significant decrease in the surface roughness was observed for a sputtering time above 10 s, which we contribute to the subsequent formation of a continuous Au layer. For a sputtering time range from 15 s to 30 s, the surface roughness increased only slightly due to the increase in the Au crystallographic grain size. For platinum, a continuous layer was readily formed at a shorter sputtering time of 5 s, due to its much smaller crystallographic grain size compared to gold^32^. Beyond a 5 s sputtering time, there was only a minor variation in the surface roughness of the deposited Pt layers. From these obtained results, for Au and Pt layers optimal sputtering times of 15 and 5 s were selected for further pattering our metal anoparticle arrays because these times yield continuous, metallic films with a low surface roughness. The thicknesses of these Au and Pt layers were ~12.5 and 4.5 nm, respectively.

### Argon ion-beam etch rates at various beam incident angles

**Fig. 5:**
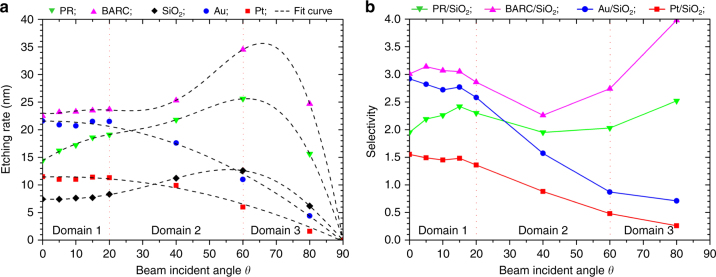
**a** Etching rate of five materials (PR, BARC, SiO_2_, Au, and Pt) as function of the beam incident angle. **b** Etching selectivity of PR, BARC, Au, and Pt with respect to SiO_2_ versus beam incident angle

There are two common problems encountered with ion-beam etching that are the strong dependence of the etching rate of materials on the beam incident angle, and redeposition of back-sputtered materials on the surface of steep features. Therefore, the etching rate of the used materials as function of the beam incident angle is needed in order to obtain an optimal etching recipe for particular structures, especially at the nanoscale. In this work, we investigated the etching rate versus the beam incident angle of five materials, i.e., SiO_2_, PR, BARC, Au, and Pt (Fig. 5a). As the beam incident angle increases from normal incidence, the etching rate of SiO_2_, PR, and BARC reached a maximum for an angle between 50° to 70°, subsequently decreasing at glancing angles. This behavior is well-known from literature^33–35^. Lee^35^ attributed this initial increase in the etching rate to an increase in the probability of collisions that results in atoms possessing momentum components directed away from the material surface. At high incident angles, the incoming ion-beam is spread over a large surface area, resulting in a decreased ion flux, and hence a reduction in the etching rate. Moreover, at glancing angles the purely elastic reflection of the primary incident ions increases significantly, leading to a sharp decrease in the etching rate. In contrast to SiO_2_, PR, and BARC, the etching rate of Au and Pt showed a maximum at the normal incidence, and the etching rate decreases with increasing incident angle. A similar behavior in the etching rate of Au and Pt as a function of the beam incident angle has been observed by Gosset et al.^36^, who attributed this to the used ion energy of 500 eV, which is rather low. Increasing the ion energy to 2–50 keV would lead to a more classic IBE behavior of these materials, in which their etching rates increase with the increasing incident angle^37^.

As shown in Fig. 5a, the etching rate of PR was much lower than that of BARC for the entire range of beam incident angles (Supplementary Figure S6). We attribute this to the difference in the molecular structure and composition of PR (i-line photoresist) and BARC (AZ BARLi)^38^. Compared to BARC, PR contains a novolac (phenol-formaldehyde) resin with aromatic rings, and a diazonaphthoquinone (DNQ) sensitizer^39^, giving the PR a much larger molecular weight than BARC. Moreover, the DNQ sensitizer acts as a dissolution restrainer for the unexposed novolac resin after baking, leading to difficulty in breaking the chemical bonds in the PR. The highest etching rate of SiO_2_ was ~12.6 nm min^−1^, which is almost three times lower than that of BARC (Table 1). We explain this large difference to the fact that SiO_2_ has much stronger covalent bonds than BARC and PR^40^, requiring a larger amount of energy for breaking the bonds. Figure 5a also shows that the maximum etching rate of Au was nearly two times higher than that of Pt, although these materials have a similar crystallographic structure (FCC), and almost the same atomic mass. The difference in the etching rate of these materials has also been reported in literature^36,41^. It can be attributed to the difference in the metallic bonding strength between Au atoms and Pt atoms, which is measured by the enthalpy of atomization. In the third series of transition metal, the enthalpy of atomization first increases with increasing number of d electrons up to six electrons per atom, i.e., the half-filled s+d band, and subsequently decreases^42^. Below six d electrons, only bonding orbitals are filled, leading to the enhancement of cohesive energy, while further filling of electrons results in the occupation of the antibonding orbitals, leading to the decrease in the cohesive energy.

**Table 1:** Etching rate of five materials (PR, BARC, SiO_2_, Au, and Pt) at various beam incident angles

Material	Etching rate (nm min^−1^)							
	0°	5°	10°	15°	20°	40°	60°	80°
PR	14.4	16.2	17.2	18.6	19.1	21.8	25.6	15.6
BARC	22.3	23.2	23.3	23.5	23.7	25.3	34.5	24.7
SiO_2_	7.4	7.4	7.6	7.7	8.3	11.2	12.6	6.2
Au	21.6	20.9	20.7	21.5	21.4	17.6	11.0	4.4
Pt	11.5	11.0	11.0	11.4	11.3	9.9	6.0	1.6

For patterning uniform metal nanoparticle arrays, a high uniformity in the nanocolumn height and the shape of the nano-sharp tips remaining during the etching process is a crucial requirement. Since the nano-sharp tips are used as an etching mask (Fig. 2), a low etching selectivity of BARC and PR with respect to SiO_2_ is needed in order to reduce their damage during the overetching of the substrate, i.e., SiO_2_. Moreover, the etching rates of Au and Pt, and their selectivities with respect to SiO_2_ need to be high enough in order to etch through the deposited metal layer and to remove the redeposited metal on the nanocolumn sidewall. Figure 5b shows the etching selectivity of PR, BARC, Au, and Pt with respect to SiO_2_ as function of the beam incident angle, separated into three domains. The etching rates of these materials and their selectivities at the beam incident angles in domain 1 (0° ≤ *θ *≤ 20°) fulfill the above-stated requirements. Therefore, in our paper, three angles within this domain were selected for further patterning arrays of Au and Pt nanoparticles, i.e., 0°, 10°, and 20°.

### Silica-supported size-tunable metal nanoparticle arrays

**Fig. 6:**
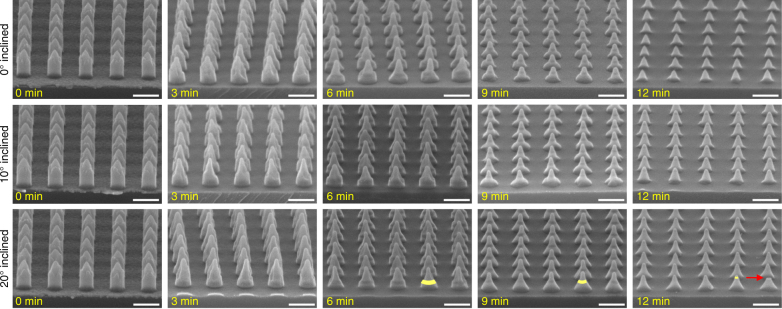
Cross-sectional HR-SEM images (tilt correction at 20°) of BARC nanocolumns inclined-etched in the IBE system at different beam incident angles versus the etching time. Overetching the Au-coated substrate resulted in Au nanoparticles supported on cone-shaped silica features, indicated by the yellow areas. The red arrow indicates the removal of a BARC nano-sharp tip caused by the breaking of the sample for SEM observation. Scale bars represent 200 nm

Figure 6 shows the etching results for periodic BARC nanocolumns patterned on Au-coated oxidized Si-wafers at three beam incident angles of 0°, 10°, and 20°. At a normal beam incidence, it was difficult to remove the Au that was redeposited on the sidewall of the BARC nanocolumns, which resulted in a relatively low uniformity of the structural geometry. Moreover, perpendicular bombardment of high energy Ar ions on the BARC nano-sharp tips leads to the rapid removal of these tips. As a result, at an etching time of 12 min, no Au nanoparticles remained on the cone-shaped silica features. Increasing the beam incident angle to 10° resulted in a faster removal of the redeposited Au, and a considerable increase in the uniformity of the structures. As can be seen in Fig. 6, the highest structural uniformity was obtained at an etching angle of 20°. We attribute this to the rapid removal of the redeposited Au, and the significant decrease in the physical bombardment of Ar ions on the BARC tips under this inclined etching angle. A low etching of the BARC nano-sharp tips leads to precise control of the fabrication of the metal nanoparticles with the etching time. Therefore, in this work, a beam incident angle of 20° was selected for further investigation on the patterning of Au and Pt nanoparticles arrays.

**Fig. 7:**
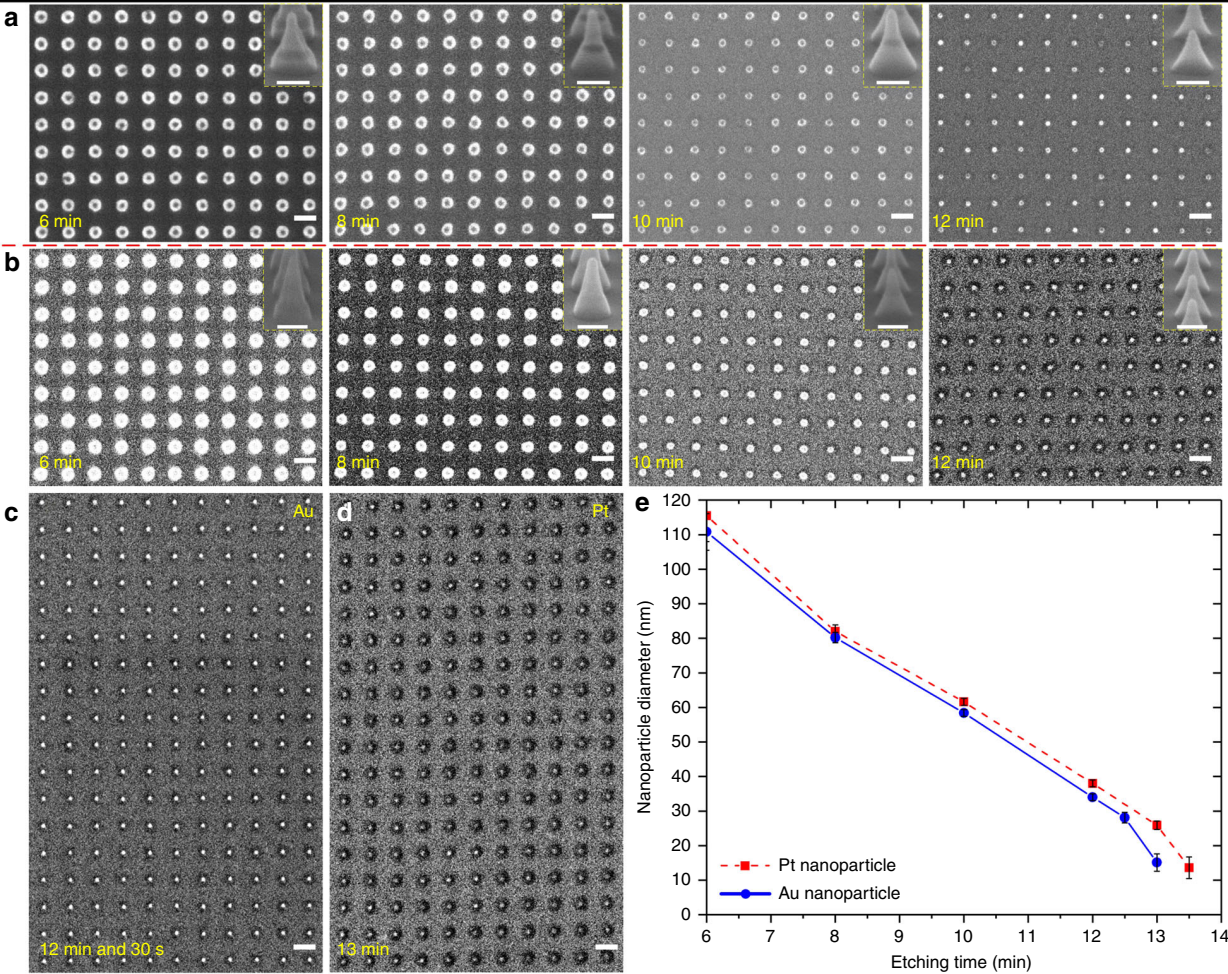
Top-view HR-SEM images of **a**,** c** Au and **b**,** d** Pt nanoparticles (bright spots) supported on cone-shaped silica features at different etching times, recorded with back-scattered electrons. The close-up cross-sectional images (scale bar: 100 nm) inserted **a** and **b** show the geometry of the structure after etching at particular etching times. The black spots in **a** indicate that the BARC nano-sharp tips remained during the etching process. **b** These BARC tips were completely removed by using oxygen plasma cleaning for 20 min at 500 W. **c**, **d** Arrays of sub-30 nm Au and Pt nanoparticles, respectively. **e** Diameter measurement of Au and Pt nanoparticles versus the etching time. Scale bars represent 200 nm

Figure 7 shows top-view HR-SEM images of arrays of Au and Pt nanoparticles supported on cone-shaped silica features after various etching times. After every etching time, it is remarkable that the BARC nano-sharp tips were preserved during the etching process with a high uniformity in the shape and the height, as can be seen in the close-up image inserted in Fig. 7a (Supplementary Figure S7). These BARC tips could subsequently be removed by using an oxygen plasma (TePla 300) at 500 W for 20 min (Fig. 7b). From Fig. 7b recorded with back-scattered electrons, we could also again confirm the continuity of the sputtered Pt layer at 5 s in the T’COathy system. The size of Au and Pt nanoparticles could be varied from the original ~110 nm to sub-30 nm by increasing the etching time up to 12 min and 30 s and 13 min, respectively (Fig. 7c,d). A high uniformity in the particle diameter was obtained for both Au and Pt nanoparticles after etching at particular etching times (Supplementary Figures S8–S9 and Table S2). It is remarkable that the Au and Pt nanoparticles remained without damage after the oxygen plasma cleaning process (Supplementary Figure S9). For an etching time of 12 min and 30 s, and 13 min, arrays of sub-30 nm Au and Pt nanoparticles were obtained with a high uniformity in particle diameter, 28.1 ± 1.5 nm and 25.9 ± 1.2 nm, over the patterned 3×3 cm^2^ areas, respectively (Supplementary Figure S10). A further increase in etching time of 30 s resulted in arrays of sub-20 nm Au and Pt nanoparticles, though with a considerable decrease of uniformity in particle diameter distribution, 15.1 ± 2.5 nm and 13.6 ± 3.1 nm, respectively (Fig. 7e and Supplementary Figure S11).

Subsequent annealing in air (TSD-12 furnace, Toma, Netherlands) of sub-30 nm Au and Pt nanoparticle arrays at 300 °C (Au) and 600 °C (Pt) for 1 h resulted in sub-20 nm Au and Pt nanoparticle arrays at a high uniformity in the particle diameter, 13.0 ± 1.6 nm and 13.2 ± 1.1 nm, respectively (Supplementary Figure S12). It is highly remarkable that these annealed sub-20 nm Au nanoparticle arrays were found to be very stable in water using a quick dump rinser for 10 min, i.e. flowing water (Supplementary Figure S13). We attribute this to the significant increase in the adhesion of Au nanoparticles with silica substrate after this annealing process^43^. Our fabrication method could also be used to pattern sub-100 nm Au nanoparticle arrays from an initially 45 nm thick Au layer. On the other hand, an initially 22.5 nm thick Pt layer resulted in the destruction of the BARC nano-sharp tips during the etching process (Supplementary Figure S14). We attribute this to the approximately two times lower etching rate of Pt versus Au and BARC, which makes the removal of the redeposited Pt on the sidewall of the BARC nanocolumns more difficult. Consequently, the BARC nano-sharp tips were removed faster than the redeposited Pt. Heating such arrays of sub-100 nm Au nanoparticles lead to the formation of sub-100 nm Au nanocrystals with facets supported on curve-shaped silica features (Supplementary Figure S15). The annealed sub-100 nm Au nanocrystal array was found to be very stable in flowing water for 10 min (Supplementary Figure S16), and could be used to study gas-bubble growth around laser-irradiated, water-immersed plasmonic nanoparticles^44^. However, we found that the non-annealed array of sub-100 nm Au nanoparticles supported on cone-shaped silica features could be directly transferred onto a piece of adhesive tape due to the insufficient adhesion of Au nanoparticles with the silica features as no metallic adhesion layer was used (Supplementary Figure S17). Further investigation of this process and the adhesion of Au with the material substrates is expected to result in better transfer over large areas. In fact, although non-annealed Au nanoparticles revealed insufficient adhesion in flowing water, we expect that their adhesion is sufficient for our intended gas-phase catalysis study.

## Conclusion

In conclusion, we successfully demonstrated a versatile top–down nanofabrication method for patterning large-scale arrays of highly ordered noble metal nanoparticles supported on oxidized silicon substrates, without the need of metallic adhesion layers. Our fabrication method combines UV-based DTL with a N_2_ plasma etching technique and an Ar ion-beam etching technique. The N_2_ plasma etching technique is used to pattern periodic BARC nanocolumns with nano-sharp tips that are used as a mask for further patterning metal nanoparticles from the metal layer deposited on SiO_2_ substrates, using an inclined Ar ion-beam etching technique. Upon applying this method to films of 12.5 nm Au or 4.5 nm Pt, we fabricated 3×3 cm^2^ arrays of Au or Pt nanoparticles supported on cone-shaped silica features at various diameters. By tuning the inclined etching time, the particle diameters could be varied from sub-30 nm to 110 nm. Annealing such sub-30 nm metal nanoparticle arrays at high-temperature resulted in sub-20 nm metal nanoparticle arrays with high uniformity in the particle diameter. By using a post-annealing process, we significantly enhanced the adhesion of Au nanoparticles with the silica substrate. The annealed Au nanoparticles were found to be very stable in flowing water.

As our fabrication method relies only on dry-etching techniques—physical bombardment with ions or atoms of nitrogen and argon—we believe that this method can be extended to pattern size-tunable nanoparticle arrays of different metal-support combinations. In case of thicker films, i.e., 100 nm Au, we have the opportunity to transfer our Au nanoparticle arrays to substrates with a stronger adhesion. With its easy and flexible operation, our fabrication method presents an enabling technique for rapidly patterning highly uniform arrays of size-tunable metal nanoparticles over large areas that can be used in biological/chemical applications, e.g., trace analyte detection^45^, dopamine sensing^46^, and protein and cell surface analyses^47,48^.

Our ongoing research focuses on integration of these fabricated arrays of silica-supported Au and Pt nanoparticles into microreactor chips. Different gas-phase chemical reactions will be conducted in these chips, for various particle size distributions, for investigating the influence of particle size on their catalytic activity and the mass transfer effect at the particle surface^2,49^.

## Electronic supplementary material

Supplementary Information
