# Supplementary material for: Large-scale fabrication of highly ordered sub-20 nm noble metal nanoparticles on silica substrates without metallic adhesion layers
Source: Microsyst Nanoeng. 2018 Apr 23;4:4. doi: 10.1038/s41378-017-0001-2 (PMC6161447; doi:10.1038/s41378-017-0001-2)
Supplement: Supplementary file 1 — Supplementary Information [file 41378_2017_1_MOESM1_ESM.docx]

Large-scale fabrication of highly ordered sub-20 nm noble metal nanoparticles on silica substrates without metallic adhesion layers

Hai Le-The, Erwin Berenschot, Roald M. Tiggelaar, Niels R. Tas, Albert van den Berg, Jan C. T. Eijkel

SI1. Uniformity measurement of fabricated BARC nanocolumn arrays

A BARC nanocolumn array of 3×3 cm^2^ fabricated on a gold coated oxidized Si-wafer was investigated. Periodic BARC nanocolumns were transferred directly at a 1:1 ratio from pre-patterned PR nanocolumns by using N_2_ plasma etching in a reactive ion etch (RIE) system (home-built TEtske machine, MESA+, NanoLab) at wafer-level, 10 mTorr, and 25 W for 8 min. The uniformity of these fabricated BARC nanocolumns was analyzed by measuring their geometric dimensions at five different selected areas (Figure S1).

Figure S2(a) shows top-view and cross-sectional HR-SEM images of the BARC nanocolumns taken at these selected areas. The cross-sectional HR-SEM images show well-defined BARC nanocolumns with highly vertical sidewall over these selected areas. The formation of nano-sharp tips was also observed, which is attributed to the physical bombardment of high energy particles during N_2_ plasma etching. The top-view HR-SEM images show a high uniformity in the diameter and periodicity of the fabricated BARC nanocolumns. The diameter distributions of the BARC nanocolumns analyzed by ImageJ software within these top-view images show only a slightly difference in the diameter variation.


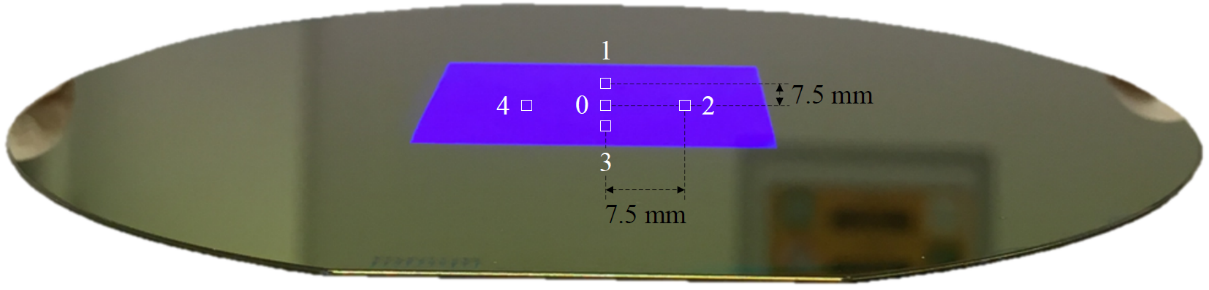


**Figure S1.** A BARC nanocolumn array (3×3 cm^2^) patterned on a Au-coated oxidized Si-wafer. Five areas indicated by white squares were selected for the uniformity measurement of the fabricated BARC nanocolumns.


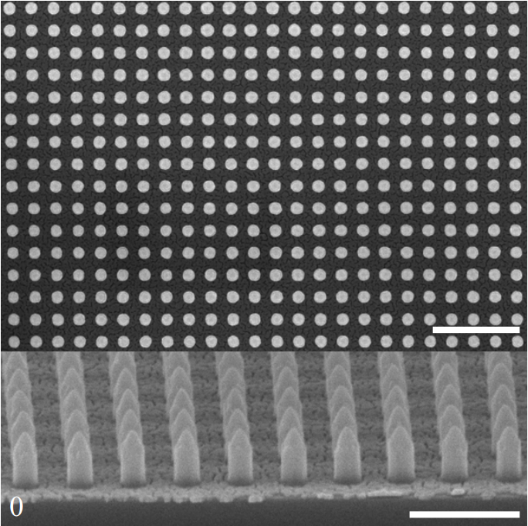

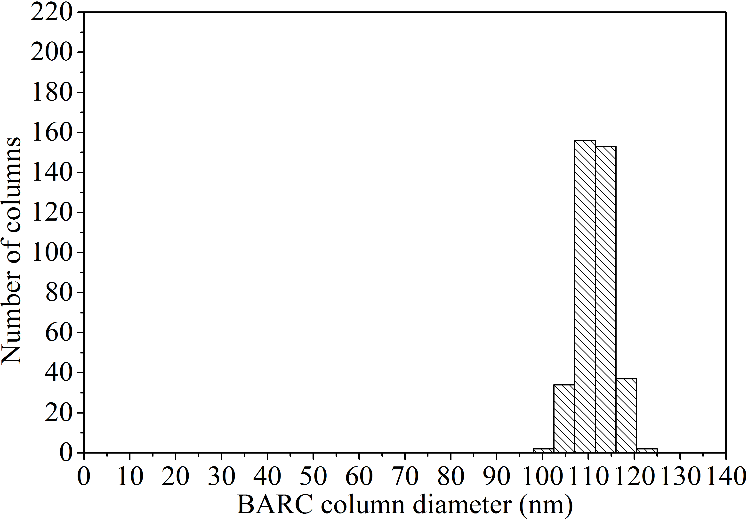


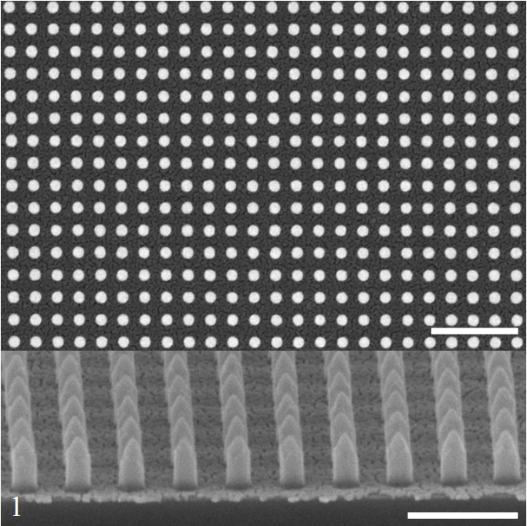

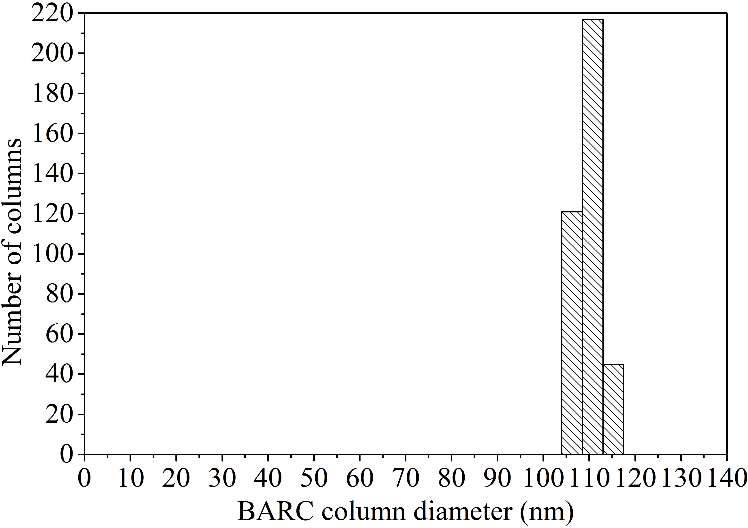


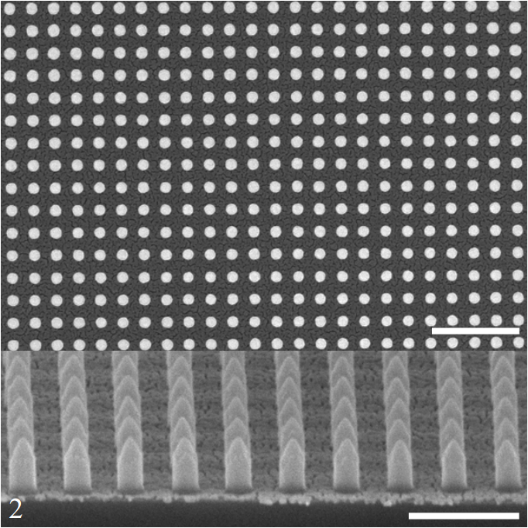

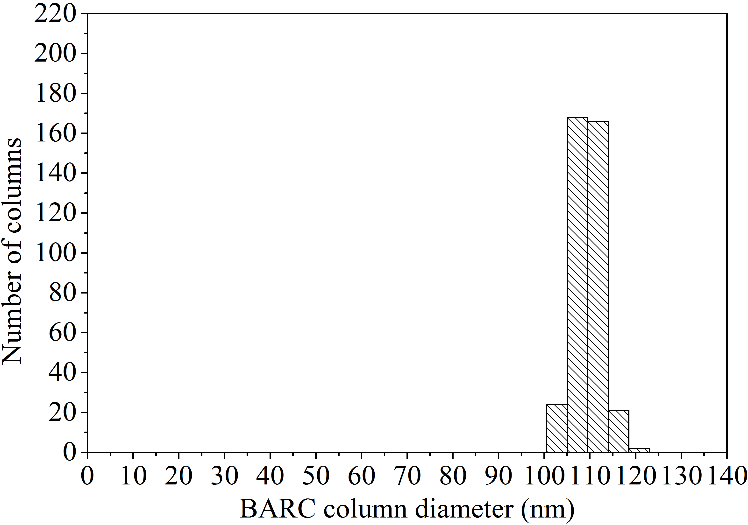


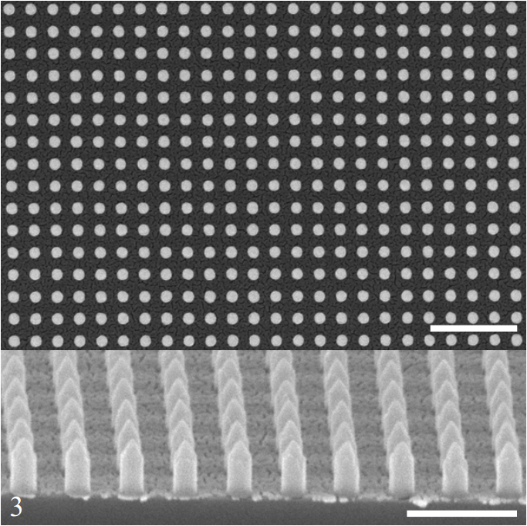

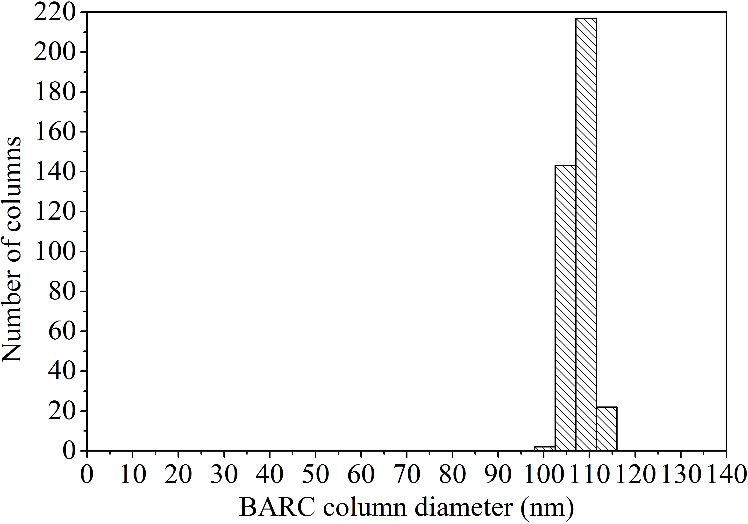


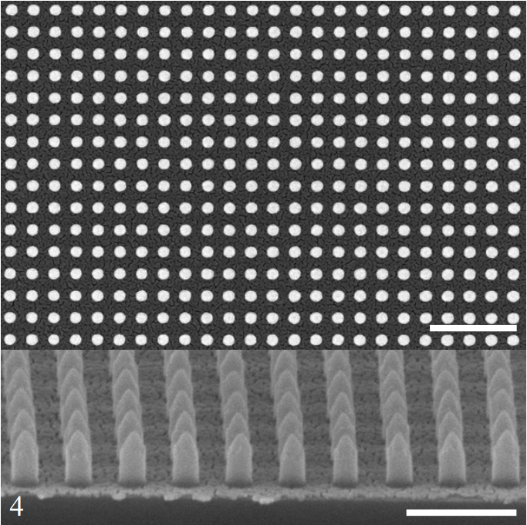

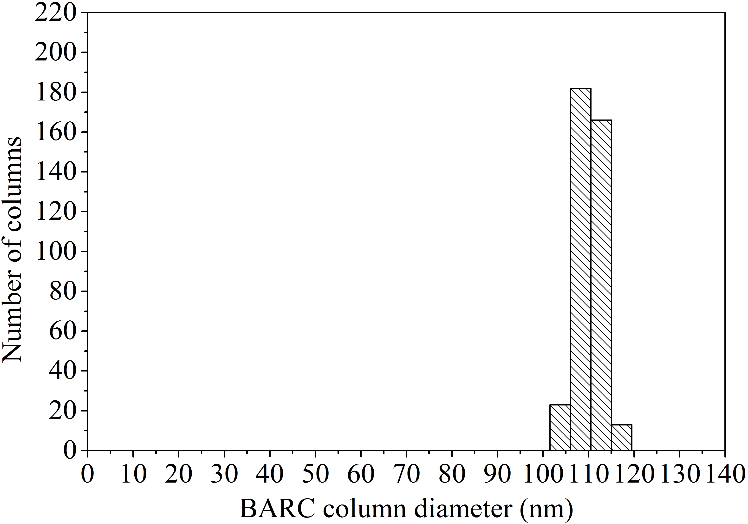


**a** **b**

**Figure S2.** **a**) Top-view (scale bar: 1 μm) and cross-sectional (scale bar: 500 nm) HR-SEM images of the fabricated BARC nanocolumns at the five selected areas, and (**b**) the corresponding column diameter distributions.

To quantitatively analyze the uniformity in diameter and height of the fabricated BARC nanocolumns, close-up cross-sectional HR-SEM images at these selected areas were taken (Figure S3). At each selected area, the average column diameter and height were calculated from at least five measured values; and the variation was calculated as the standard deviation of these measured values (Table S1).


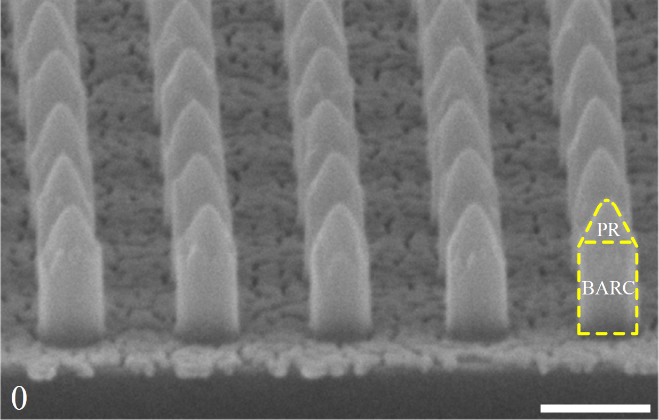


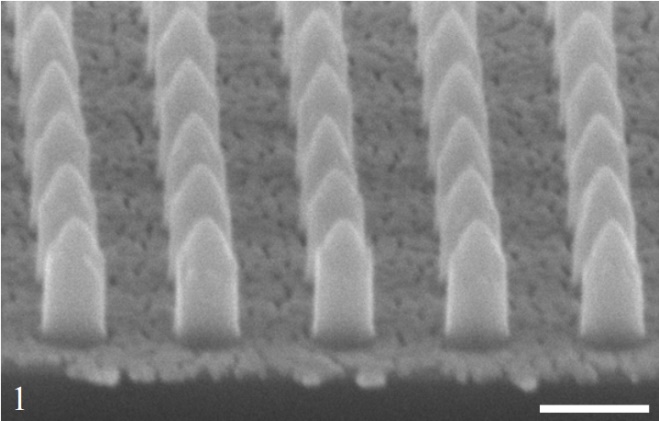

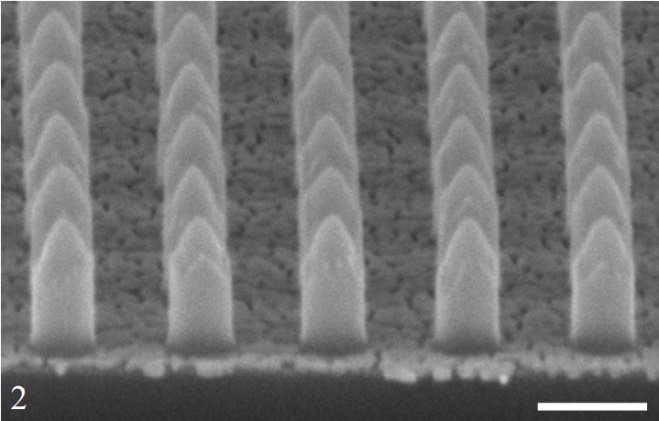


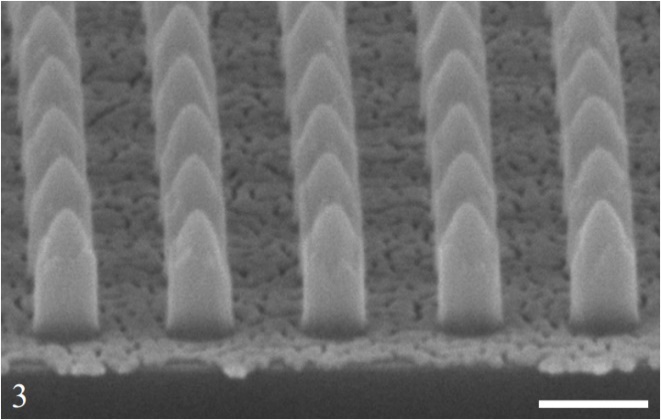

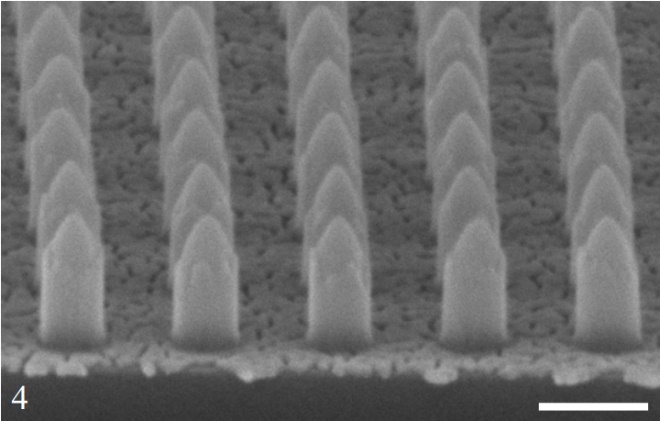


**Figure S3.** Close-up cross-sectional HR-SEM images of the fabricated BARC nanocolumns at the five selected areas. Scale bars represent 200 nm.

**Table S1.** Uniformity measurement in diameter and height of the fabricated BARC nanocolumns in Figure S3. The variation was calculated as the standard deviation.

| Area | 0 | 1 | 2 | 3 | 4 |
| --- | --- | --- | --- | --- | --- |
| Column diameter (nm) | 110.2±1.7 | 110.6±1.4 | 110.8±1.3 | 110.7±0.7 | 110.6±1.3 |
| Column height (nm) | 251.4±1.5 | 249.8±1.9 | 250.6±1.6 | 251.6±0.8 | 250.8±1.1 |

SI2. Surface roughness measurement of sputtered Au and Pt layers

All sputtering processes were conducted by using an ion-beam sputtering system (home-built T’COathy system, MESA+, NanoLab) at a pressure of 6.6×10^-3^ mbar, and 200 W. The thickness of the deposited metal layers was varied by adjusting the sputtering time. Figures S4 and S5 show atomic force microscopy (AFM) and HR-SEM images of the deposited Au and Pt layers at different sputtering times, respectively. For both Au and Pt layers, the surface roughness (Ra) measured in AFM images increased with the increasing layer thickness. We attribute this to an increase in the Au or Pt crystallographic grain size as the layer thickness increases.^[S1]^ In addition, the small crystallographic grain size in the Pt layers leaded to a much lower surface roughness compared to the Au layers.

**
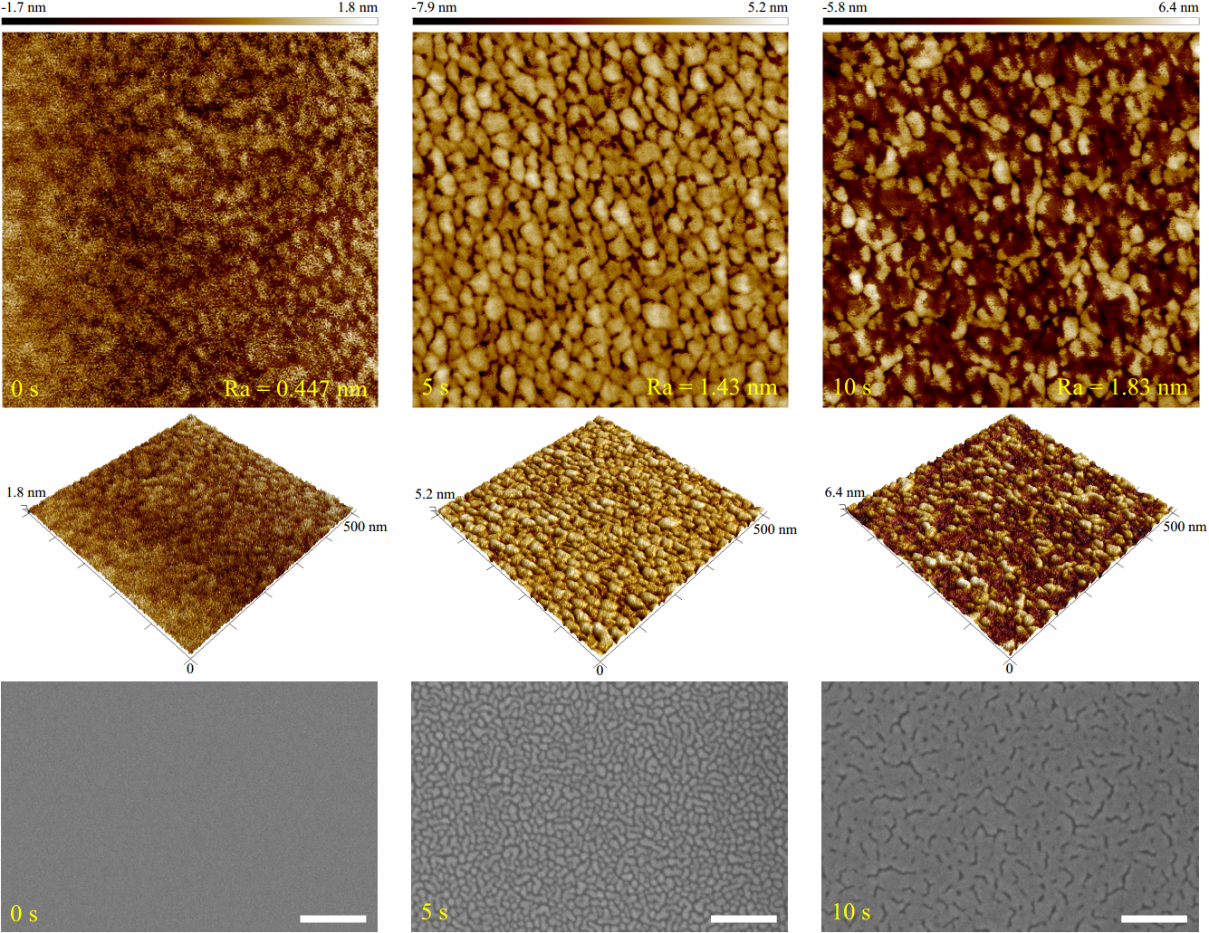
**

**
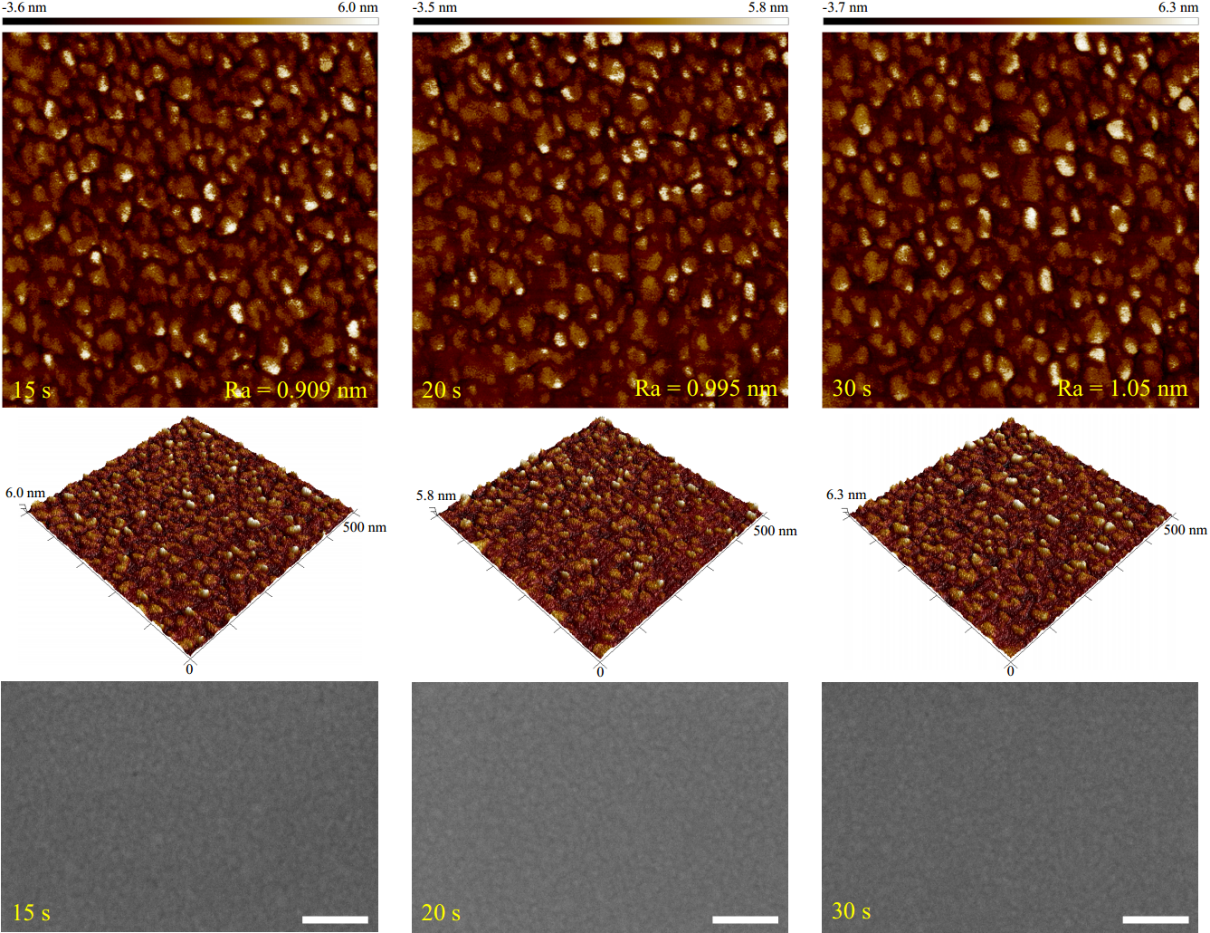
**

**Figure S4.** AFM (scan field 500×500 nm^2^) and top-view HR-SEM (scale bar: 200 nm) images of Au layers deposited at different times on oxidized Si substrates.


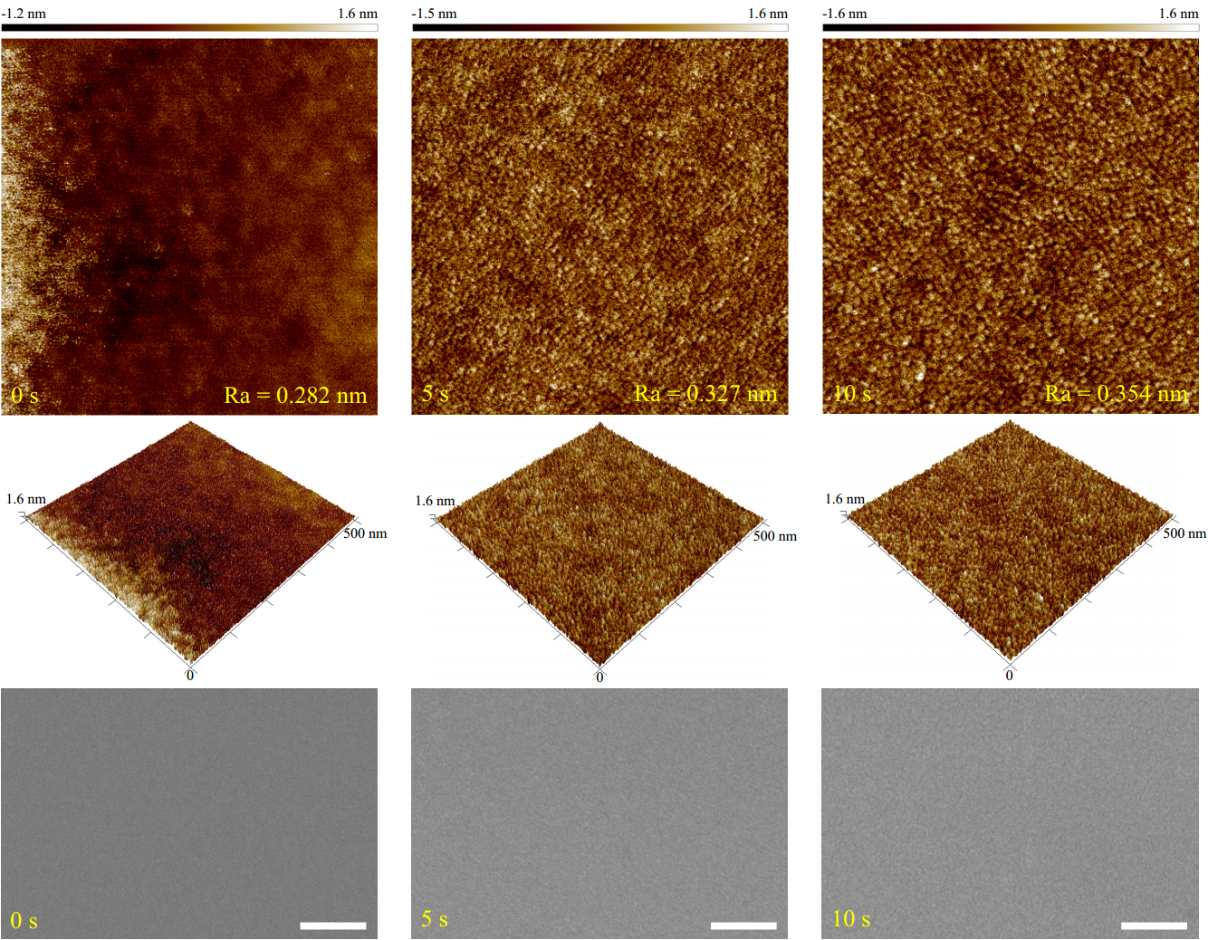


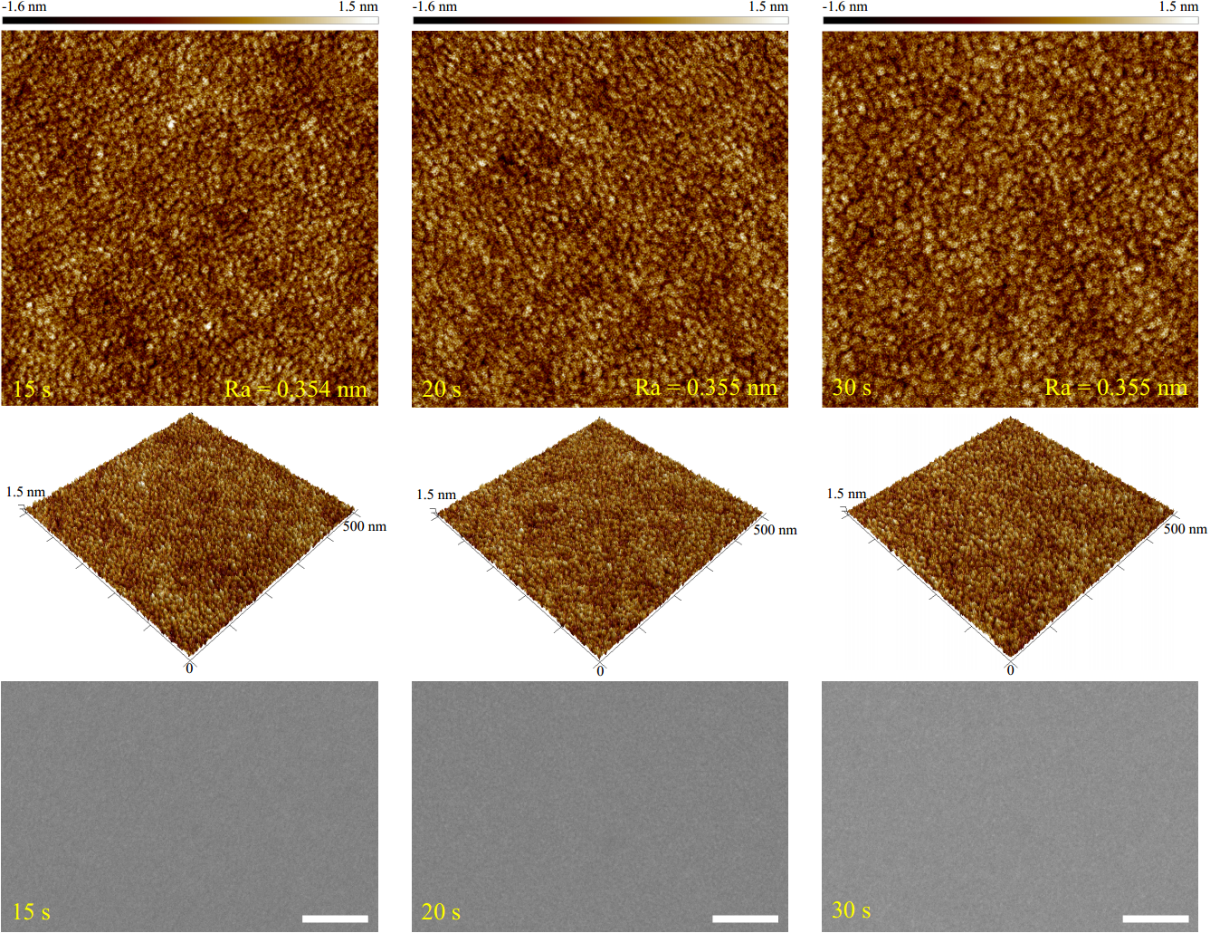


**Figure S5.** AFM (scan field 500×500 nm^2^) and top-view HR-SEM (scale bar: 200 nm) images of Pt layers deposited at different times on oxidized Si substrates.

SI3. Etching of PR and BARC layers at various beam incident angles

PR layers (299.3±1.8 nm) and BARC layers (245.8±1.5 nm) were spin-coated on oxidized Si-wafers at 2000 rpm for 45 s, followed by baking at 90℃ and 185℃, respectively. The etching rate at each etching angle was determined by comparing the initial layer with the layer after etching for 3 min. The thicknesses of the PR and BARC layers were determined from the images taken using a HR-SEM (FEI Sirion microscope) at a 5 kV acceleration voltage and a spot size of 3 (Figure S6).


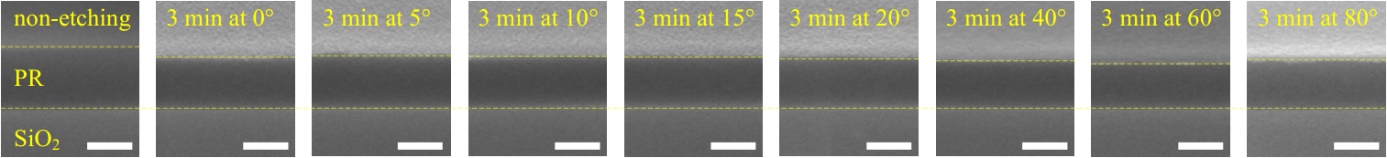


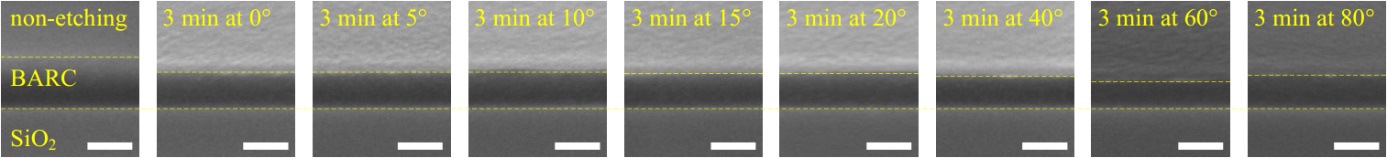


Figure S6. Cross-sectional HR-SEM images of PR and BARC layers etched in an IBE system (Oxford i300) at various beam incident angles for 3 min. The etching was conducted at 5 sccm Ar, 300 eV, and 50-55 mA. Scale bars represent 200 nm.

SI4. Patterning Au or Pt nanoparticle arrays using inclined Ar ion beam etching

Au and Pt nanoparticle arrays were fabricated by etching the periodic BARC nanocolumns patterned on Au or Pt -coated SiO_2_ substrates in the IBE system at a beam incident angle of 20° (Figure S7). It is remarkable that the BARC nano-sharp tips remained during the etching process with a high uniformity in the shape and the height. The size of the Au and Pt nanoparticles can be varied by changing the etching time, as shown in Figure S8. A high uniformity in the particle diameter was obtained for both Au and Pt nanoparticles after etching at particular etching times (Table S2).


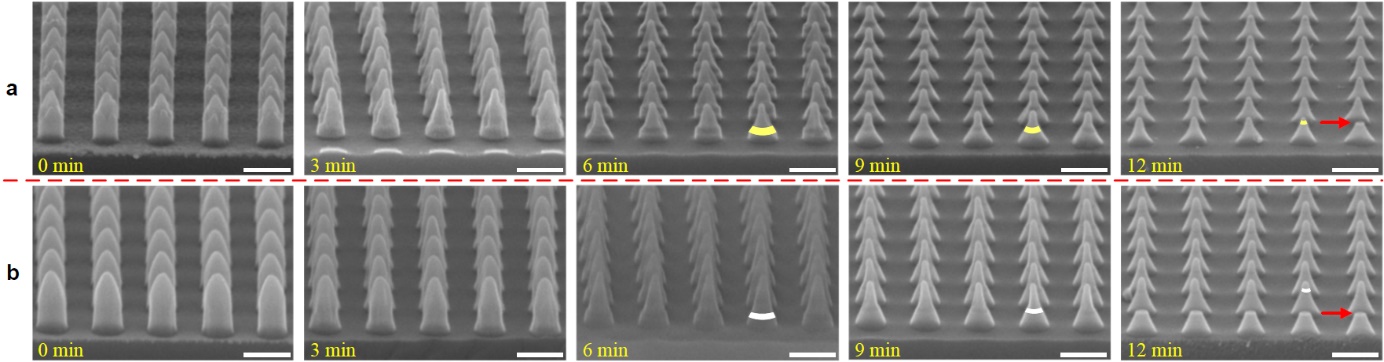


Figure S7. Cross-sectional HR-SEM images of BARC nanocolumns inclined etched in the IBE system at different etching times. The etching was conducted at 5 sccm Ar, 300 eV, 50-55 mA, and at a beam incident angle of 20°. Over-etching the metal-coated SiO_2_ substrate resulted in (a) Au and (b) Pt nanoparticles supported on cone-shaped silica features, indicated by the yellow and white areas, respectively. The BARC nano-sharp tips remained during the etching process. The red arrows indicate the removal of the BARC nano-sharp tips caused by the breaking of samples for SEM observation. Scale bars represent 200 nm.

**Table S2.** Uniformity measurement in the diameter the fabricated Au and Pt nanoparticles in Figure S8. The variation was calculated from the standard deviation of at least five measured values.

| Etching time (min) | 6 | 8 | 10 | 12 |
| --- | --- | --- | --- | --- |
| Au nanoparticle diameter (nm) | 110.8±5.3 | 80.2±1.5 | 58.4±1.2 | 34.3±1.1 |
| Pt nanoparticle diameter (nm) | 115.5±7.5 | 82.0±1.9 | 61.6±1.3 | 38.0±1.0 |


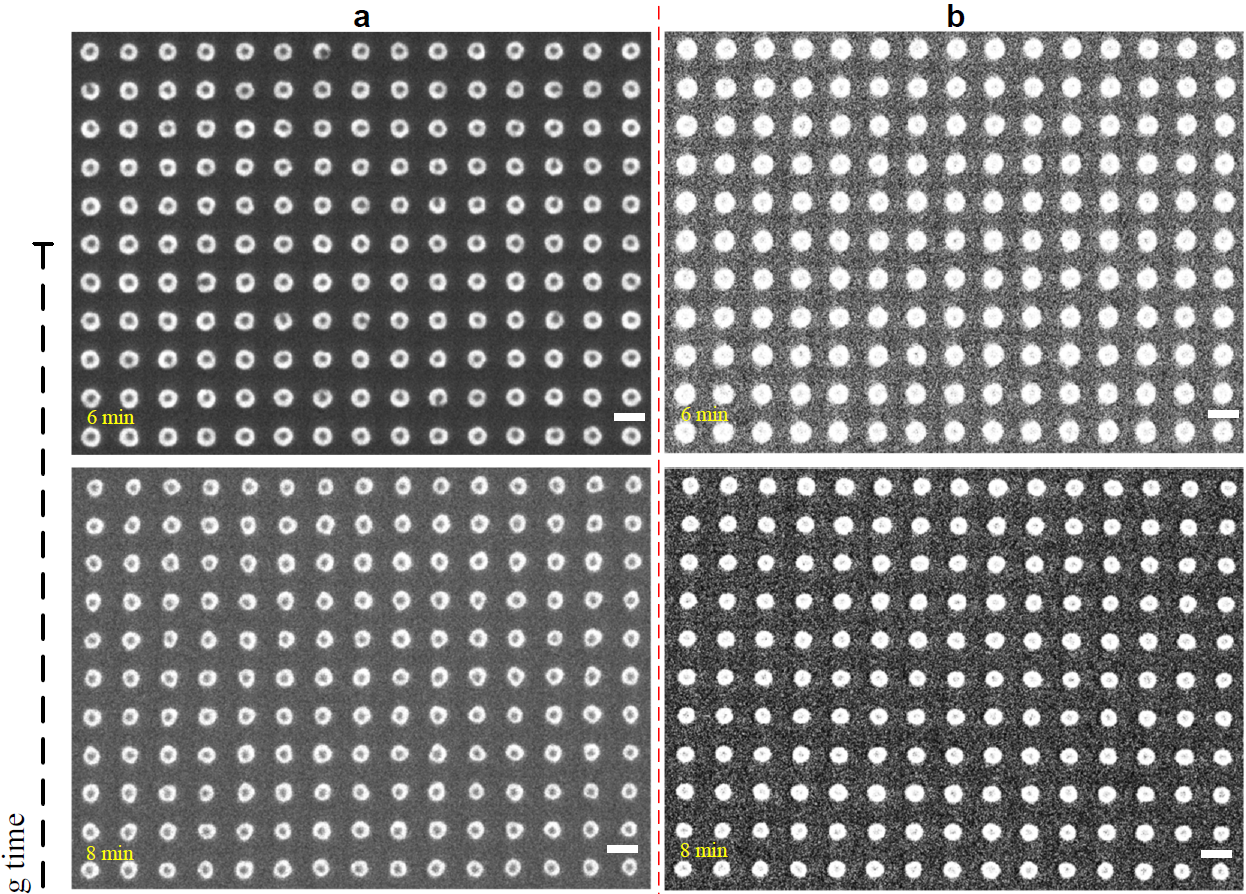

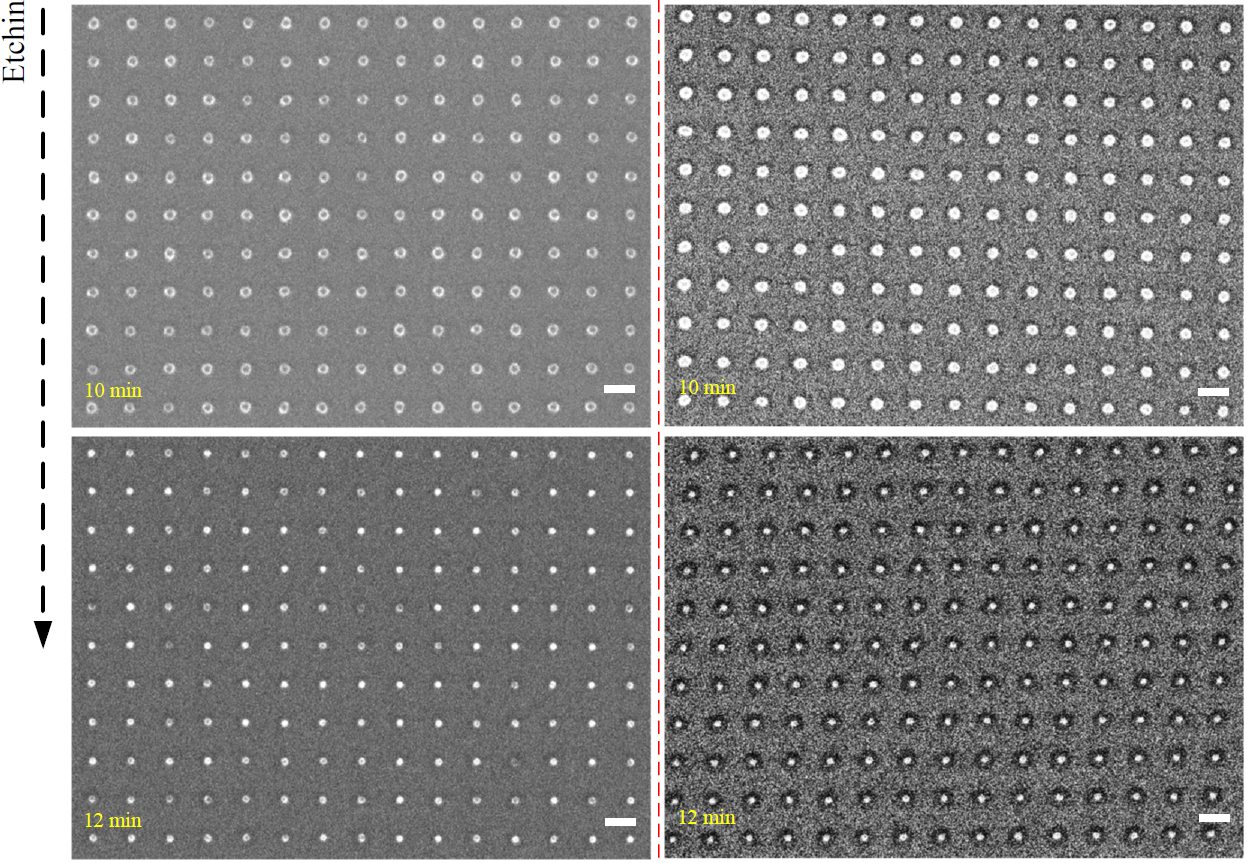


**b**

**a**

Figure S8. Top-view HR-SEM images of (a) Au and (b) Pt nanoparticles (bright spots) supported on cone-shaped silica features at different etching times, recorded with back-scattered electrons. The Au and Pt nanoparticle arrays were fabricated by etching the periodic BARC nanocolumns supported on Au or Pt -coated SiO_2_ substrates in the IBE system at a beam incident angle of 20°. The black spots in (a) indicate that the BARC nano-sharp tips remained during the etching process. These BARC tips were removed by using oxygen plasma cleaning for 20 min at 500 W (b). Scale bars represent 200 nm.

Figure S9 shows the diameter measurement of Au and Pt nanoparticles after inclined etching in the IBE system for 12:30 min (12 min and 30 s) and 13 min, respectively. Sub-30 nm Au and Pt nanoparticles were obtained with a high uniformity in the particle diameter, 28.1±1.5 nm and 25.9±1.2 nm, over the patterned 3×3 cm^2^ areas, respectively.


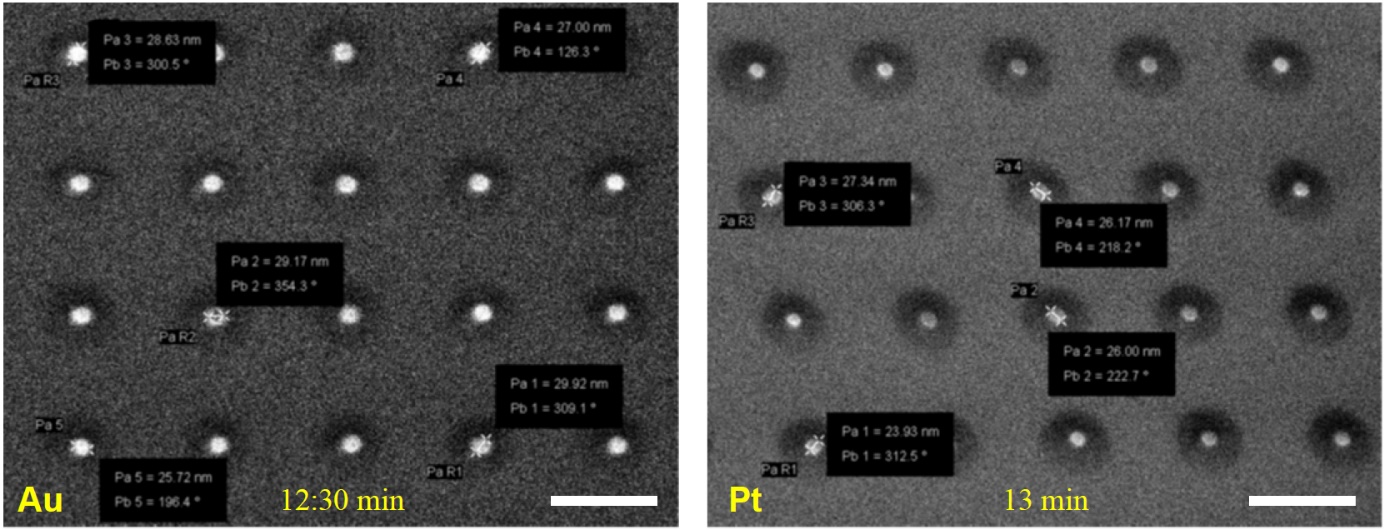


Figure S9. Close-up top-view HR-SEM images of sub-30 nm Au and Pt nanoparticles (bright spots) supported on cone-shaped silica features at the etching time of 12:30 min (12 min and 30 s) and 13 min, respectively. The BARC tips were removed by using oxygen plasma cleaning for 20 min at 500 W (TePla 300 plasma etcher). Scale bars represent 100 nm.

Figure S10 shows the top-view HR-SEM images at the selected areas of a fabricated array of sub-30 nm Au nanoparticles supported on the cone-shaped silica features, which has a high uniformity in the particle diameter.


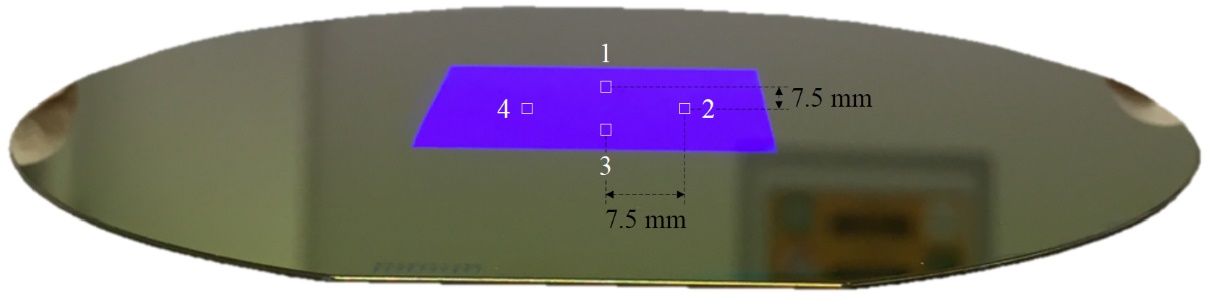

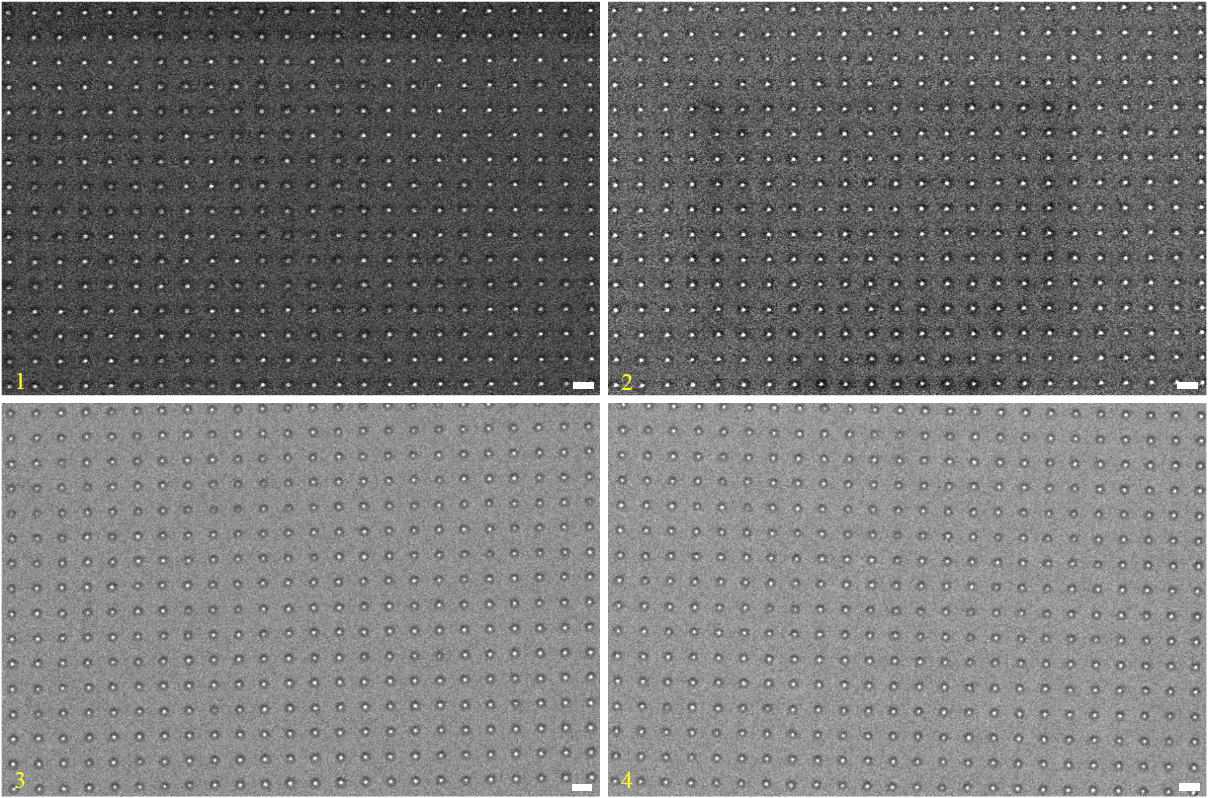


Figure S10. Top-view HR-SEM images at the selected areas of a fabricated array of sub-30 nm Au nanoparticles supported on the cone-shaped silica features, etched for 12 min and 30 s. Scale bars represent 200 nm.

Increasing the etching time with 30 s, sub-20 nm Au and Pt nanoparticles were fabricated, though at a considerable decrease of the uniformity in the particle size distribution, 15.1±2.5 nm and 13.6±3.1 nm, respectively (Figure S11). In addition, several BARC nano-sharp tips were removed at these etching times, leading to direct exposure of the Au and Pt nanoparticles to high energy Ar ions. As a result, the Au and Pt nanoparticles were etched away from those cone-shaped silica features.


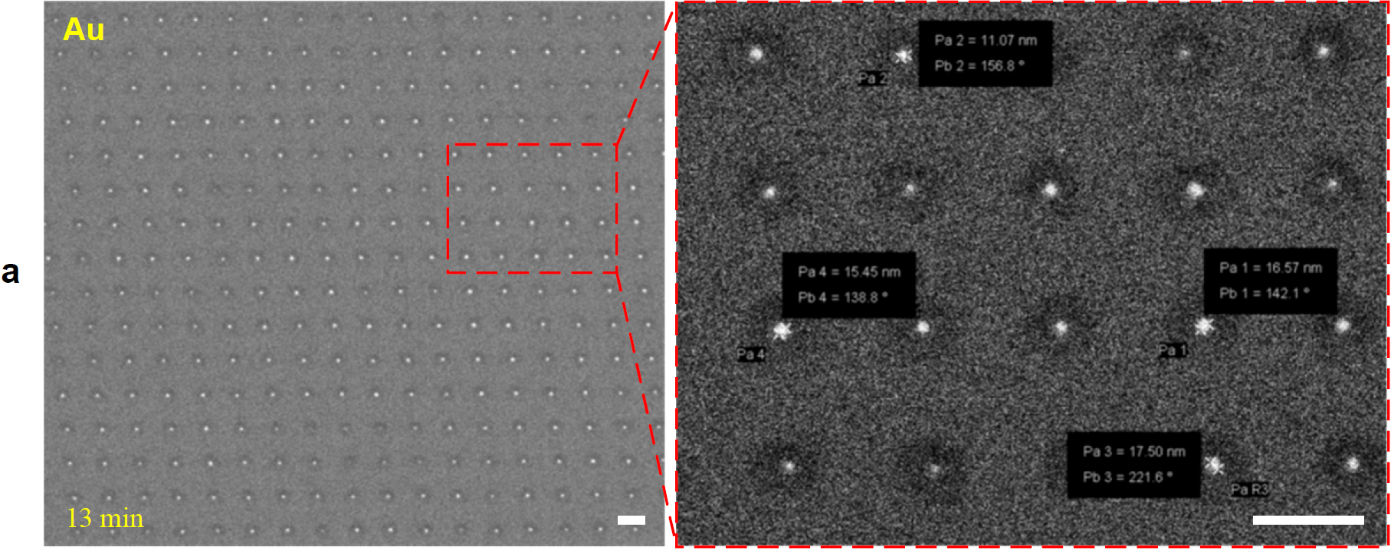


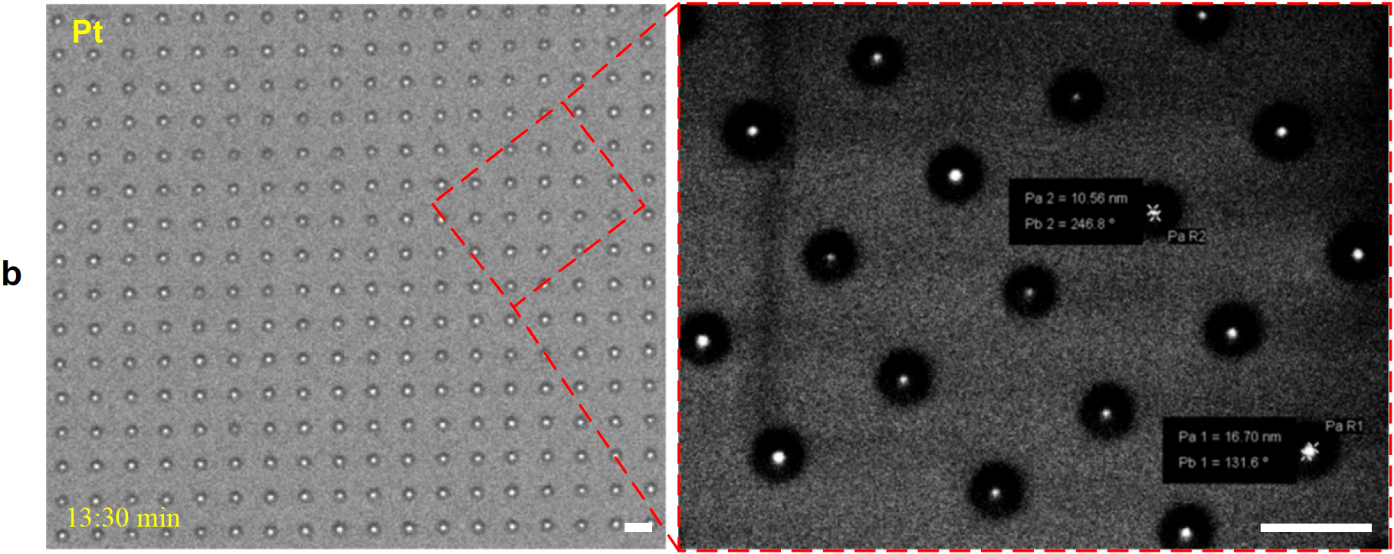


Figure S11. Top-view HR-SEM images (scale bar: 200 nm) with close-up images (scale bar: 100 nm) of sub-20 nm (a) Au and (b) Pt nanoparticles (bright spots) supported on cone-shaped silica features at the etching time of 13 min and 13:30 min (13 min and 30 s), respectively.

Subsequent annealing of sub-30 nm Au and Pt nanoparticle arrays at 300℃ and 600℃ for 1 h resulted in sub-20 nm Au and Pt nanoparticle arrays with a high uniformity in the particle diameter, 13.0±1.6 nm and 13.2±1.1 nm, over the patterned 3×3 cm^2^ areas, respectively (Figure S12).


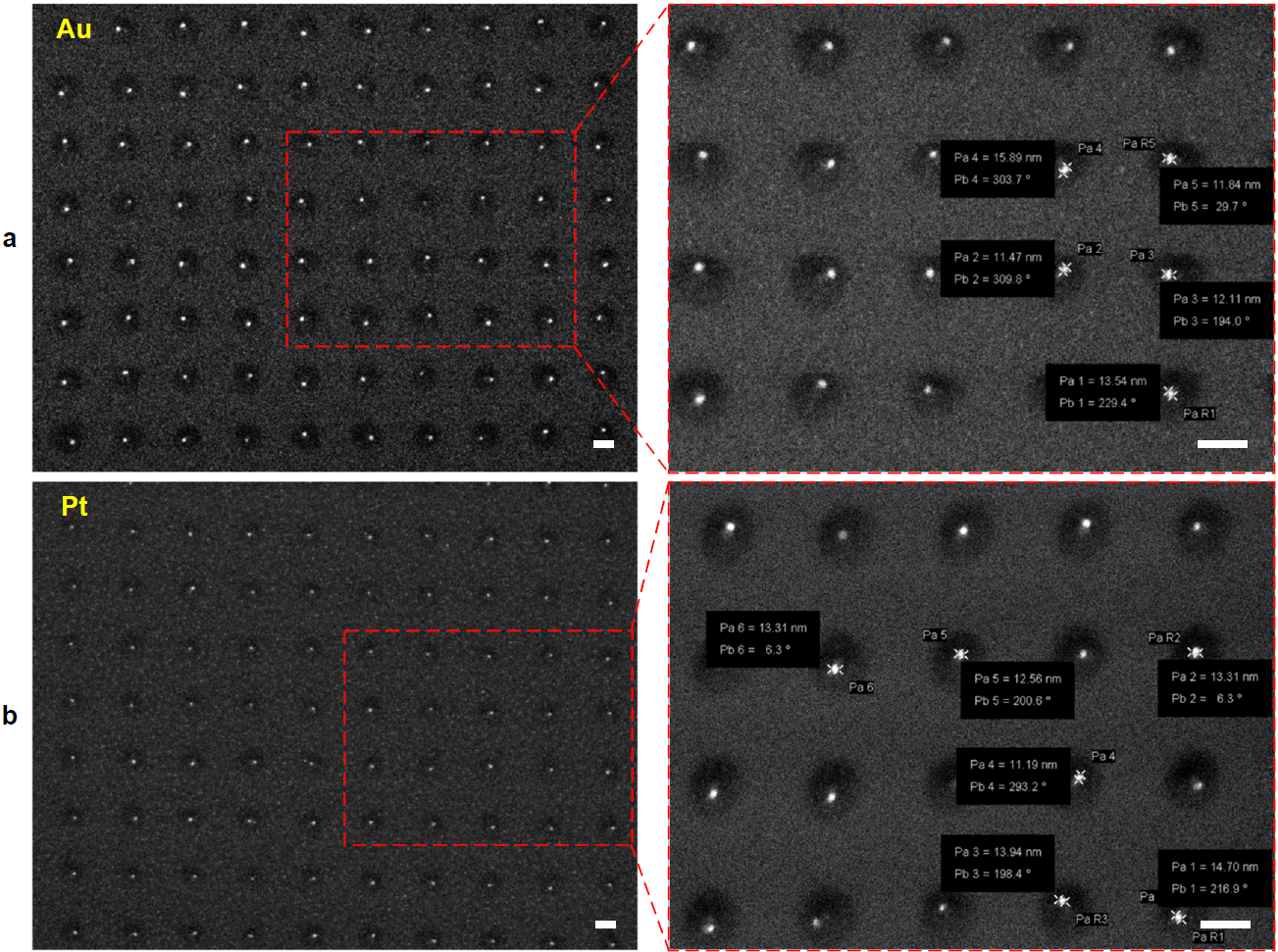


Figure S12. Top-view HR-SEM images with close-up images of sub-20 nm (a) Au and (b) Pt nanoparticles (bright spots) supported on cone-shaped silica features after heating at 300℃ (Au) and 600℃ (Pt) for 1h, respectively. Scale bars represent 100 nm.

SI5. Adhesion investigation of the fabricated Au nanoparticle arrays

An adhesion test for two fabricated arrays of Au nanoparticles supported on the silica features was conducted. An array of sub-30 nm Au nanoparticles supported on cone-shaped silica features, and an array of sub-20 nm Au nanoparticles fabricated by annealing the sub-30 nm Au nanoparticle array at 300℃ for 1 h were chosen for this adhesion verification. These arrays were rinsed with deionized (DI) water using a quick dump rinser for 10 min, and subsequently checked with the HR-SEM (Figure S13). It is highly remarkable that the annealed sub-20 nm Au nanoparticle arrays were found to be very stable in water, whereas the non-annealed sub-30 nm Au nanoparticles did not show good adhesion.


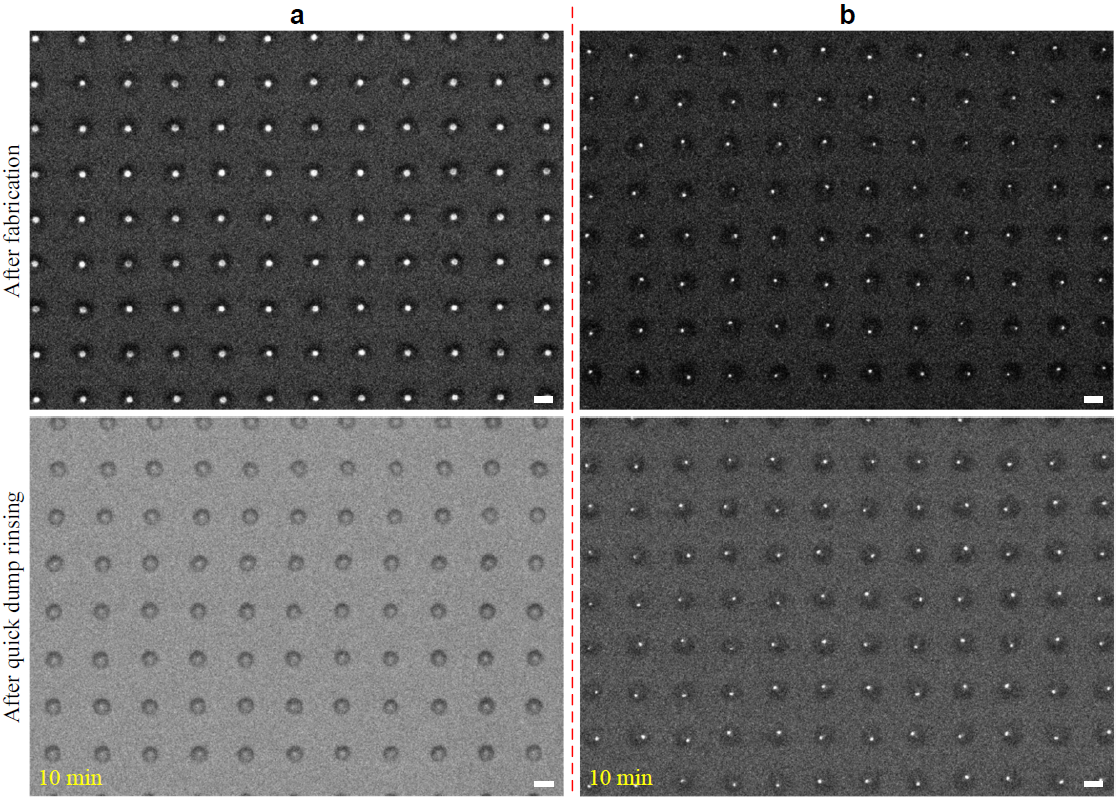


Figure S13. Top-view HR-SEM images of (a) sub-30 nm Au nanoparticles and (b) sub-20 nm Au nanoparticle arrays before and after water rinsing in a quick dump rinser for 10 min.

SI6. Inclined etching BARC nanocolumns patterned on thick deposited Au and Pt layers

Thick layers of Au (45.0 nm) or Pt (22.5 nm) were sputtered directly on the oxidized Si-wafers using the T’COathy system. Periodic BARC nanocolumns were patterned on these wafers, and subsequently etched in the IBE system at different etching times. The etching was performed at 5 sccm Ar, 300 eV, 50-55 mA, and at a beam incident angle of 20°. As can be seen in Figure S14, the BARC nano-sharp tips remained during the etching process in case of a thick Au layer, whereas they were completely destroyed in case of a thick Pt layer. We attribute this to the low etching rate of Pt, which is approximately two times less than that of Au and BARC. Therefore, it was difficult to get rid of the Pt redeposition on the sidewall of BARC nanocolumns. Consequently, the BARC nano-sharp tips were removed faster than the redeposited Pt.


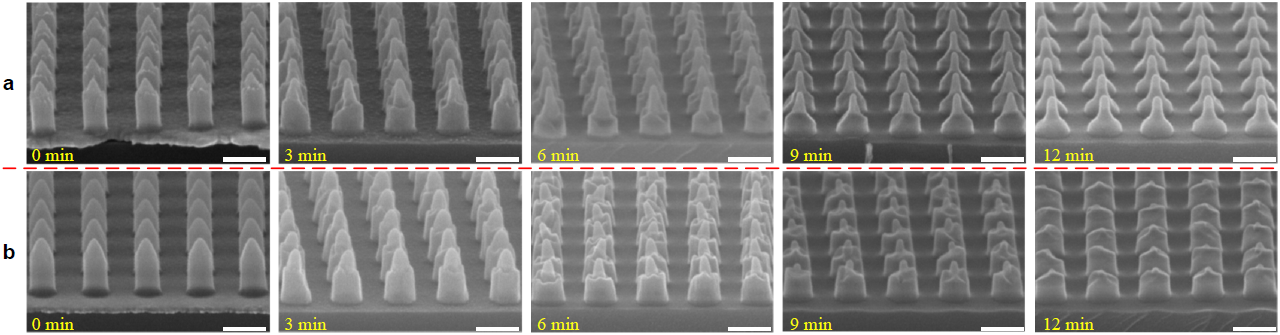


Figure S14. Cross-sectional HR-SEM images of BARC nanocolumns inclined etched in the IBE system at different etching times. These BARC nanocolumns were patterned on thick deposited layers of (a) Au (45.0 nm) and (b) Pt (22.5 nm). The etching was conducted at 5 sccm Ar, 300 eV, 50-55 mA, and at a beam incident angle of 20°. Scale bars represent 200 nm.

After an etching time of 9 min, an array of Au nanoparticles fabricated using a 45 nm thick Au layer (Figure S14a) was heated in air from room temperature to 1100℃, and passively cooled down. Before the heating treatment, the BARC nano-sharp tips were removed by using oxygen plasma cleaning for 20 min at 500 W (TePla 300 plasma etcher). During heating at high temperature, the Au nanoparticles and the cone-shaped silica features were dewetted to form sub-100 nm Au nanocrystals supported on curve-shaped silica features (Figure S15). This fabricated sub-100 nm Au nanocrystal array was also found to be very stable in water rinsing for 10 min (Figure S16).


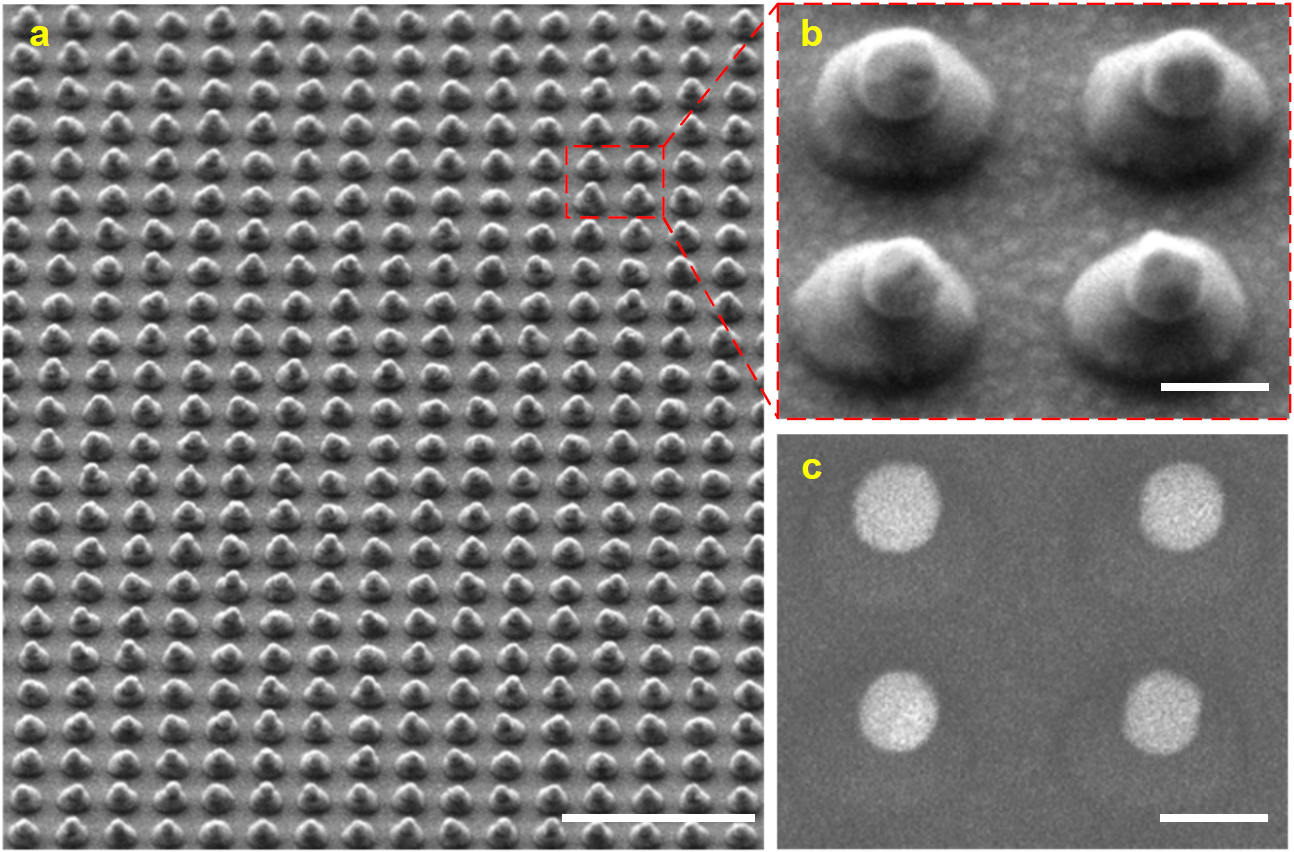


Figure S15. a) Cross-sectional HR-SEM image (scale bar: 1 μm) of sub-100 nm Au nanocrystals supported on curve-shape silica features. b) A close-up image and (c) an image recorded with back-scattered electrons (scale bar: 100 nm) indicate the Au nanocrystals (bright spots).


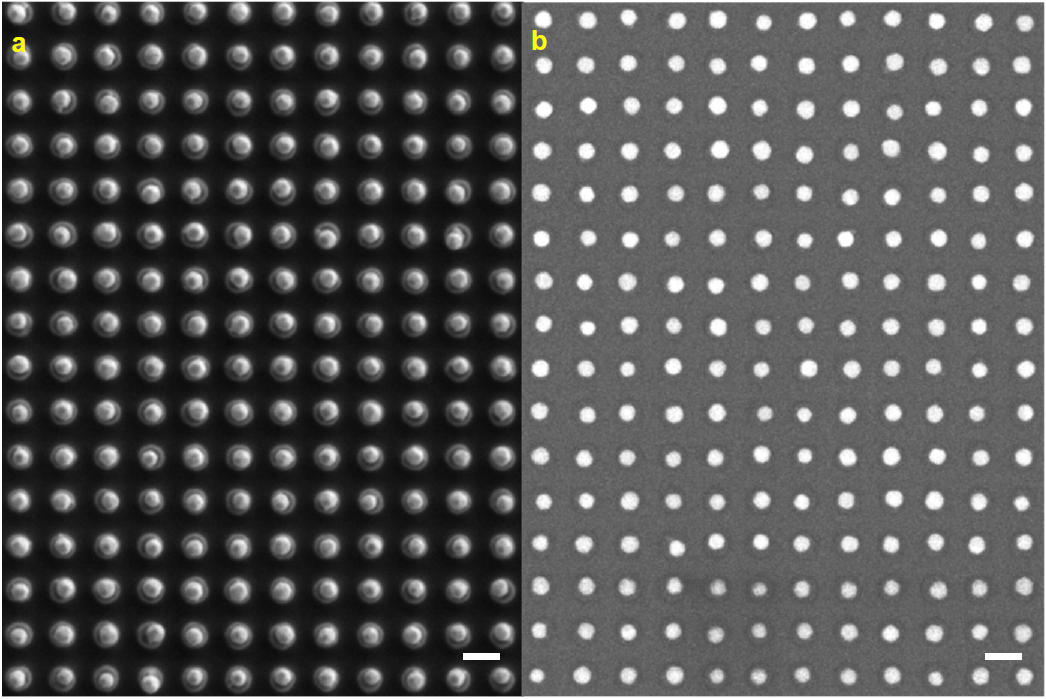


Figure S16. a) Top-view HR-SEM images of sub-100 nm silica-supported Au nanocrystals, after water rinsing in a quick dump rinser for 10 min. b) Image recorded with back-scattered electrons indicates the Au nanocrystals (bright spots). Scale bars represent 200 nm.

SI7. Transferring a Au nanoparticle array onto adhesive tape

Figure S17 shows an array of sub-100 nm Au nanoparticles supported on a piece of adhesive Scotch tape. This array was directly transferred from an array of sub-100 nm Au nanoparticles supported on cone-shaped silica features (Figure S14a) due to the insufficient adhesion of Au nanoparticles with the silica features, as no metallic adhesion layer was used.


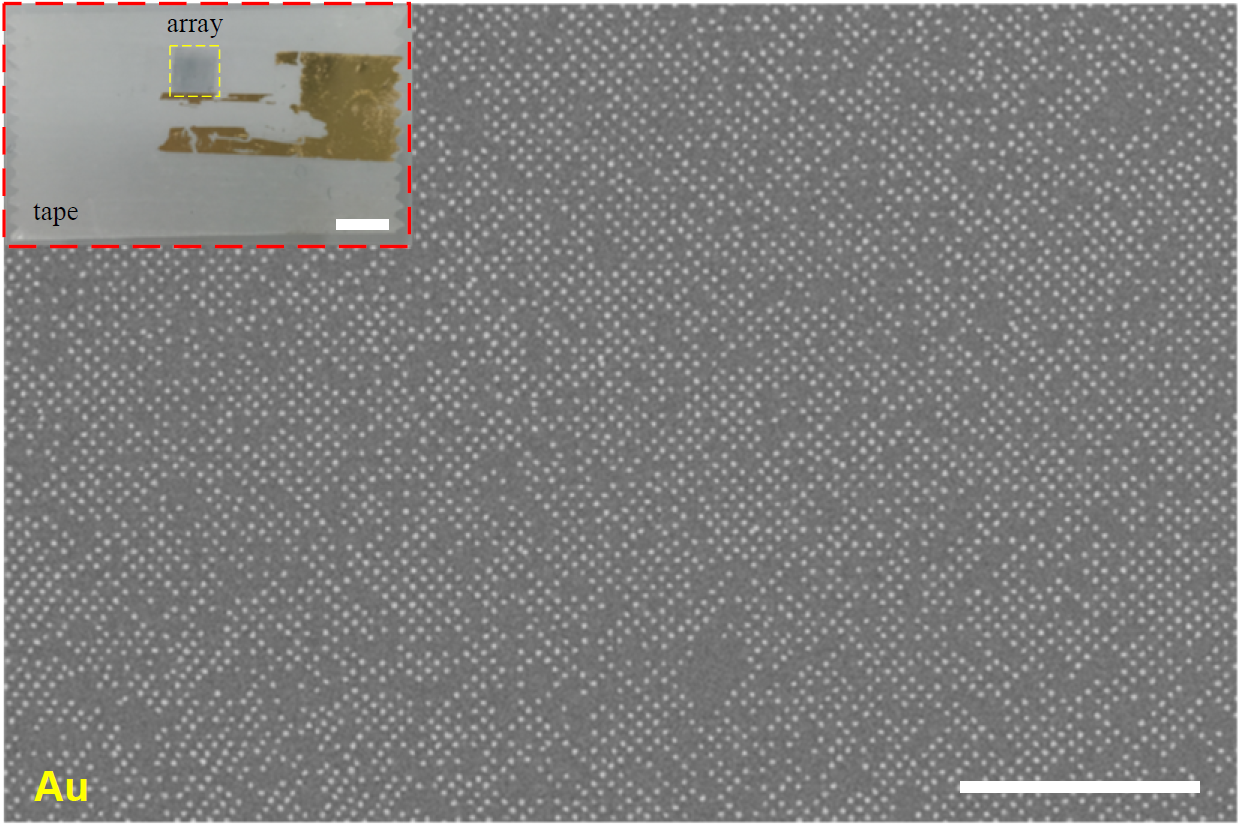


Figure S17. Top-view HR-SEM image (scale bar: 5 μm) of sub-100 nm Au nanoparticles supported on a piece of adhesive tape. The inserted optical image shows this Au nanoparticle array (yellow square), which was directly transferred from an array of Au nanoparticles supported on cone-shaped silica features (scale bar: 5 mm).

References

1. L. Melo, A. R. Vaz, M. C. Salvadori, M. Cattani, *JMNM.* 2004, *20-21*, 623.
